# Robust optical flow algorithm for general single cell segmentation

**DOI:** 10.1371/journal.pone.0261763

**Published:** 2022-01-14

**Authors:** Michael C. Robitaille, Jeff M. Byers, Joseph A. Christodoulides, Marc P. Raphael

**Affiliations:** Materials Science and Technology Division, U.S. Naval Research Laboratory, Washington, DC, United States of America; Sun Yat-Sen University, CHINA

## Abstract

Cell segmentation is crucial to the field of cell biology, as the accurate extraction of single-cell morphology, migration, and ultimately behavior from time-lapse live cell imagery are of paramount importance to elucidate and understand basic cellular processes. In an effort to increase available segmentation tools that can perform across research groups and platforms, we introduce a novel segmentation approach centered around optical flow and show that it achieves robust segmentation of single cells by validating it on multiple cell types, phenotypes, optical modalities, and *in-vitro* environments with or without labels. By leveraging cell movement in time-lapse imagery as a means to distinguish cells from their background and augmenting the output with machine vision operations, our algorithm reduces the number of adjustable parameters needed for manual optimization to two. We show that this approach offers the advantage of quicker processing times compared to contemporary machine learning based methods that require manual labeling for training, and in most cases achieves higher quality segmentation as well. This algorithm is packaged within MATLAB, offering an accessible means for general cell segmentation in a time-efficient manner.

## Introduction

It is hard to state how important live-cell microscopy has been for our understanding of biology. Currently, researchers are afforded a humbling window into the sheer complexity of cell behavior thanks to advancements in both the quality and diversity of optical modalities available for live-cell microscopy [1]. Great strides have been made on the front-end to obtain both higher quality data and larger amounts of it. However, the field has quickly realized a serious issue on the back-end—viable methods to actually analyze the imagery in meaningful and robust ways are often lacking [2, 3]. In terms of live-cell imaging, this analysis often means segmenting the cell through time lapse imagery, with the “holy grail” being robust segmentation applicable to both labeled and label-free cells to control for perturbations to the cells observed [4–6]. Indeed, the symptoms of this problem are readily apparent as biologists are so focused on the difficult task of ensuring accurate experimental design and execution, that often times little bandwidth is left over for analyzing the exquisite data strenuously collected. As a result, many complex biological functions are often over simplified—the fluid-like motions of the cell membrane during migration are treated like a rigid body by tracking the cell *via* a nucleus, or the dynamic process of cell adhesion is measured by the projected cell area at a single time point are common examples.

Such data is not left on the table willingly, but rather done so because it is a *hard* problem. This problem has garnered an immense amount of interest towards solving [7], which, in turn ironically causes its own problem: researchers already overwhelmed with the complexity of the data they have painstakingly collected are oftentimes inundated by the sheer number of purported solutions to their problems. In our own experience, many hours can be invested before realizing a well-intentioned published segmentation method is inadequate, impractically complex with regards to large numbers of tunable parameters, or requires an inordinate amount of time to manually train, with less than satisfactory results when applied to your own data set. In this regards, there is a research gap of robust segmentation techniques that can be applied across cell lines, platforms and laboratories. It is perhaps unsurprising then that cell segmentation *via* a laborious manual tracking approach is still considered the gold standard.

Great advances have indeed been made in cell segmentation [7–9], with methods falling into two general categories: image processing and parameter optimization approaches, or machine learning and deep learning approaches. Image processing methods have been historically more popular and employ a variety of techniques to distinguish cells from their background based on contrast/intensity characteristics of a single image (e.g. intensity thresholding, feature extraction, etc.). However, many methods follow an apparent common trend—they perform well only when applied to the type of imagery they were designed for [2, 3], or they require extensive re-optimization for each set of experimental imagery. Machine learning and deep learning have recently become appealing avenues for a more general method of cell segmentation [4, 10–13]. Many methods require initial supervision through manually labelled cells to teach the algorithms to correctly classify cells later on in an unsupervised matter. A draw back with these methods is that the manual training process is often time consuming, although great strides have been made to ease the burden of training [14, 15]. However, it remains an open question as to how extensive the library must be in order to achieve robust segmentation capabilities [16, 17], and can often result in users having to re-train algorithms for each data set, or even multiple times within a single data set if the imagery characteristics change across the experiment (i.e. sidewall effects). Deep learning techniques autonomously extract thousands, or even millions of parameters from the imagery, but in doing so leave users with a lack of interpretability with regards to why the algorithm performed adequately or failed. Thus, there is a significant gap for segmentation methods that are relatively easy to implement and interpret but are robust across cell types/optical modalities/data sets from various researchers (see S1 Fig in S1 File).

A viable, accessible solution for robust segmentation is to exploit aspects that are present in all live-cell microscopy, regardless of the type of optical modality and subsequent generated imagery: cellular motion and dynamic morphology. Indeed, nearly all current methods utilize the image intensity (*I)* as a function of position (x, y) within in a single image, disregarding information as to how this intensity function varies with time (*t*) and re-evaluating each image as if they are completely unrelated to the previous or next. This strikes us as a rather incomplete strategy in terms of robustness—when one considers the sheer diversity of live-cell imagery, the one unifying characteristic is that live-cell microscopy is dynamic with respect to time.

Here, we show that by leveraging the fact that live cells are morphologically dynamic, optical flow-based segmentation algorithms can offer quite robust and simple means to segment single cells from time-lapse imagery. Optical flow-based methods have been previously employed to quantify live-cell imagery, typically in the context of movement of fluorescently tagged proteins within a given cell [18–20], or tagged cells within tissues [21]. Optical flow has also been incorporated into deep learning architectures for analysis of medical imagery [22]. However, we are aware of exceptionally few cases in which optical flow is utilized for label-free cell segmentation [23], and are aware of no general (i.e. span multiple optical modalities) methods. To address this research gap, we introduce a label-free cell segmentation technique based on optical flow, and show that leveraging cellular movement via optical flow is an appealing strategy that accurately segments single cells across cell types, phenotypes, optical modalities, resolution and environments with relatively few parameters required to manually optimize. This robust algorithm is computationally inexpensive, simple to use, and packaged within MATLAB (see S1 File), offering accessibility and interpretability into dynamic single cell behavior oftentimes unavailable to typical cell biology labs.

## Methods

The foundation of the label-free segmentation algorithm outlined here is optical flow, in which the spatial changes in image intensity *I* (x, y) between two consecutive time frames is quantified to characterize relative movement. The underlying principle is that at every pixel a displacement is estimated that maps the image at time frame *t* to the image at time frame *t* + *Δt*. This assumes that while pixels can move in x and y, their net intensity *I*(x, y) remains constant, or changes negligibly between consecutive frames. More formally this can be written out as:

$$I\;\left(x,\;y,\;t\right)\approx I\;(x+u,\;y+v,\;t+\Delta t)$$ (1)

Where *u* and *v* are the local displacements of the local image regions at *x* and *y* after time Δ*t*. Another way to think of it is the pixels in image *t* are pushed to form the pixels in image *t* + Δ*t* by the optical flow field. Applying the chain rule to the right hand side of (1) [24]:

$$\frac{u}{\Delta t}\frac{\partial I}{\partial x}+\frac{v}{\Delta t}\frac{\partial I}{\partial y}+\frac{\partial I}{\partial t}=0$$ (2)

In which $\frac{u}{\Delta t}$ and $\frac{v}{\Delta t}$ are the optical flow in *x* and *y*, respectively. Eq (2) is known as the optical flow constraint equation, and the flow field calculated from it is then used as a means to estimate which pixels correspond to a cell in a given image: pixels in which there is no movement between consecutive frames do not generate much displacement and can be considered background, while pixels in which larger displacements are generated can be considered a cell, given that certain criteria are met. This approach does not depend on any particular image characteristic in *I*(*x*, *y*), but rather how *I*(*x*, *y*) changes between consecutive frames, and thus enables highly robust cell segmentation without the need for labels.

Optical flow is a general term for a variety of strategies that try to measure relative intensity displacement between consecutive images, and methods are optimized to compute either sparse or dense flow fields for either small or large object displacements. For instance, rigid objects that do not undergo shape changes between consecutive frames (i.e. cars captured on traffic cameras) are well suited for methods which employ sparse flow fields, such as Lucas-Kanade [25]. For imagery of deformable objects that may change shape between consecutive frames (for instance, a cell with filopodia extending or retracting), it is necessary to employ a dense flow field method that estimates flow within objects as well as around the perimeter. Similarly, objects that may make relatively large displacements between consecutive frames are best dealt with methods that utilize pyramidal multi-scale resolution techniques. Considering that the movement of cells can vary significantly between cell type or experimental set up and lead to relatively large cell displacements between consecutive frames, we have found the Farnebäck method to work exceptionally well for estimating flow fields of live-cell imagery.

**Fig 1:**
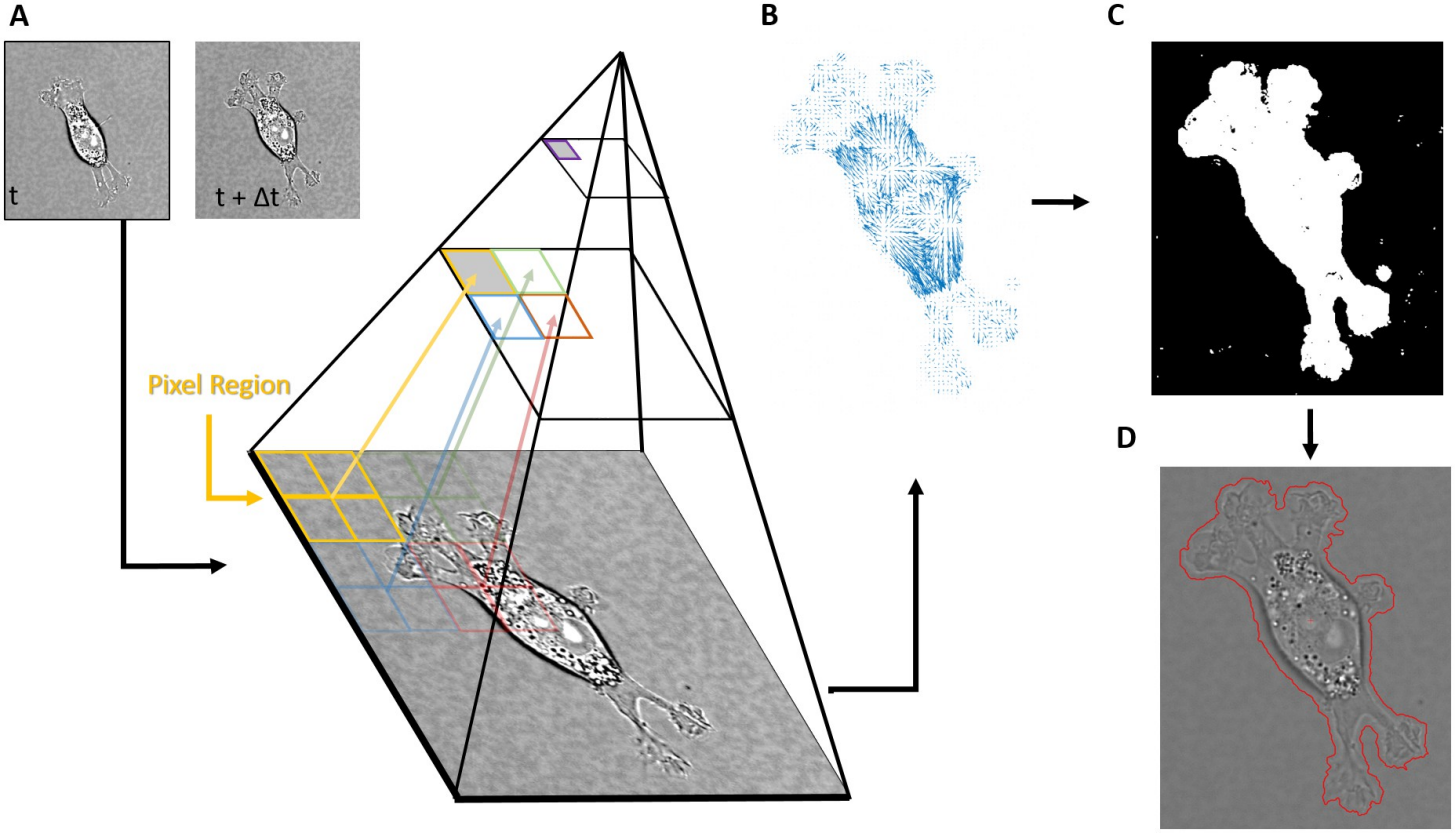
Overview of the segmentation algorithm. A) Two consecutive image frames are used to calculate the optical flow via the Farnebäck algorithm, which utilizes a multi-level resolution pyramid to estimate intensity displacements between the two frames. B) Once the flow field is calculated, a threshold Th is applied to the magnitude of the individual flow vectors, leaving only flow vectors with a magnitude above Th. C) The pixels associated with the remaining flow field form a binary mask to estimate where the cell is in the image. D) The mask is closed and filled to create the segmented perimeter of the cell.

A detailed description of the Farnebäck method is given elsewhere [26], but the key elements are 1) the use of multi-level resolution pyramid and 2) the use of quadratic polynomials to quantify the intensity of pixel regions. Fig 1A depicts this resolution pyramid, in which a grouping of four pixels are averaged to form a single pixel at the next resolution level, with the higher pyramid levels corresponding to lower resolutions of the imagery. At the lowest resolution level, regions of pixels (i.e. 5x5 square) are fitted with a quadratic polynomial weighted towards the center of the region for each time frame. It is assumed that a single displacement can describe the transformation of the fitted polynomial region at frame *t* to the fitted polynomial region at frame *t* + Δ*t*, and this displacement is iteratively calculated. The final displacement is a 2D vector that describes the flow of intensity in one region between consecutive images, and is then used as *a priori* knowledge for the fitting of quadratic polynomials at the next pyramid level below it (higher resolution). The displacement is then recalculated based on polynomial fits of newer, higher resolution regions, and in turn is used as *a priori* knowledge for the pyramid level below it, and so on. The end result is a 2D vector field that characterizes the optical flow at the true resolution of the image that is quite resilient to noise, deformable objects, and large displacements. Once the flow field is calculated at the highest resolution, a manually selected threshold is then applied to the magnitude, shown in Fig 1B. All pixels corresponding to a flow value below the threshold are considered background, while pixels with a flow value above the threshold are segmented as cells. The remaining pixels are those that exhibit significant optical flow and represent moving cells, and are subsequently grouped together and filled to create a binary mask (Fig 1C) that can then be used to segment cells from their background (Fig 1D).

**Fig 2:**
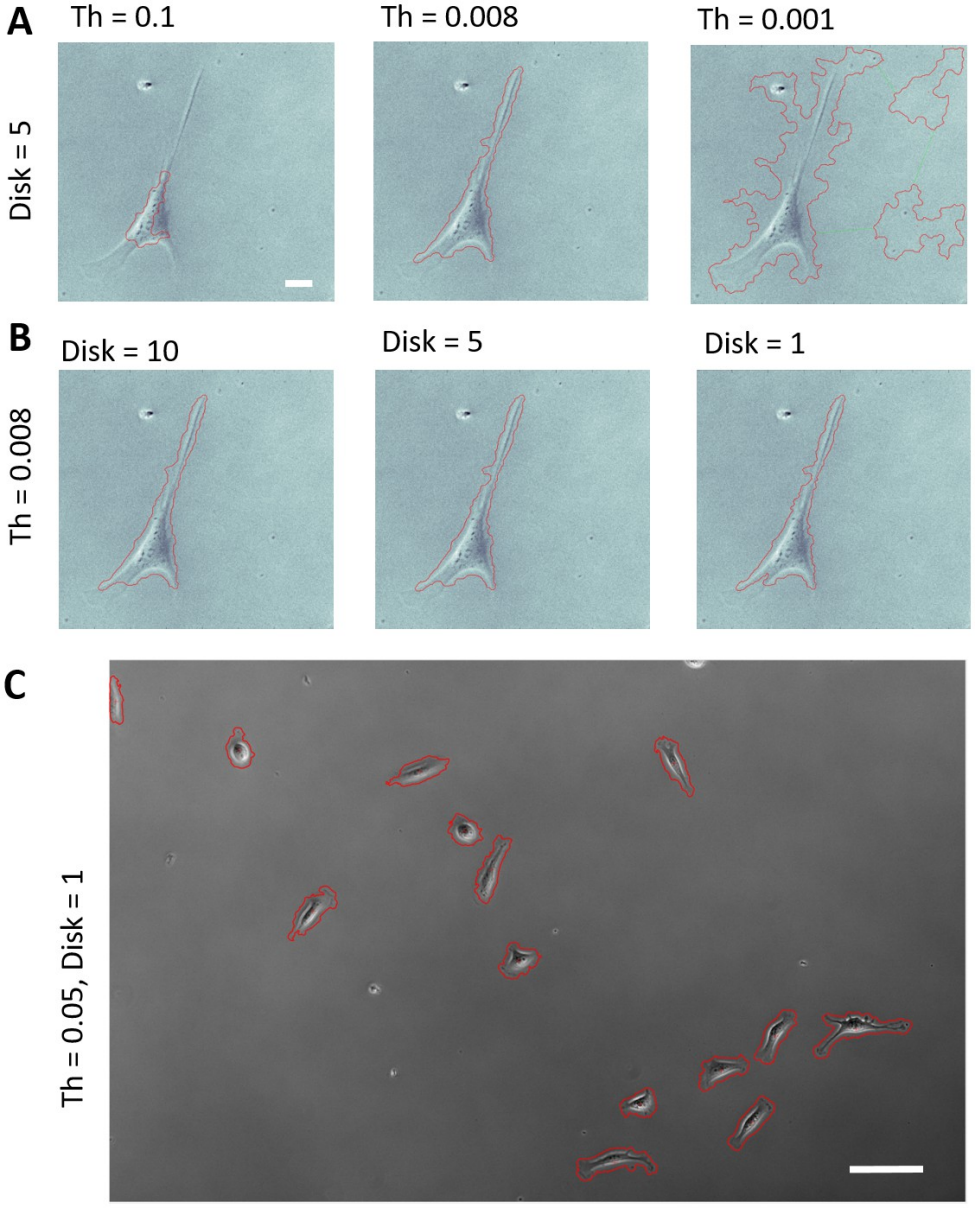
The effect of optical flow parameters on segmentation of a single Hs27 fibroblast under 10x phase contrast microscopy. A) The optical flow threshold, Th, can lead to over- or under-segmentation if not optimized, and is typically the only parameter that needs to be optimized for a given data set. Scale bar 20μm B) The smoothing disk of the segmented cell effects the perimeter, but often times does not drastically alter the accuracy of the segmentation itself. C) A larger field of view of MBA-MD-231 cells under 10x phase showing a single value of Th = 0.05 can adequately segment cells under a variety of morphologies and motion characteristics. Scale bar 50μm.

The optical flow algorithm has a single primary parameter that requires manual optimization for label-free cell segmentation: the optical flow vector magnitude threshold (Th). Additionally, two secondary computer vision parameters, a size filter, and to a lesser extend smoothing disk can be altered depending on the imagery. Each parameter is intuitive and takes little time to optimize for a given data set. High Th values only segment areas in which large changes in pixel intensity due to object motion (i.e. flow), and too high of Th values can lead to an underestimation of cell area (Fig 2A). Similarly, too low of Th values can pick up smaller intensity changes outside of a cell due to stochastic fluctuations in cameras or experimental artifacts and can often times lead to an over estimation of cell area. The size filter simply prohibits the segmentation of objects smaller than the entered value to eliminate the segmentation of cellular debris, precipitates, or other objects common *in-vitro*. Finally, the smoothing disk is a standard morphological operation for filling small holes and smoothing the segmented perimeter of the cell (Fig 2B). Oftentimes, the size filter and smoothing disk are robust with regards to cell types or optical modalities, leaving only the threshold (Th) to optimize manually for end-users. For a given experimental set up/cell type, a single Th value is often robust enough to capture a variety of different cell morphologies within a single field of view, as is shown in Fig 2C.

## Results

### Validation

**Fig 3:**
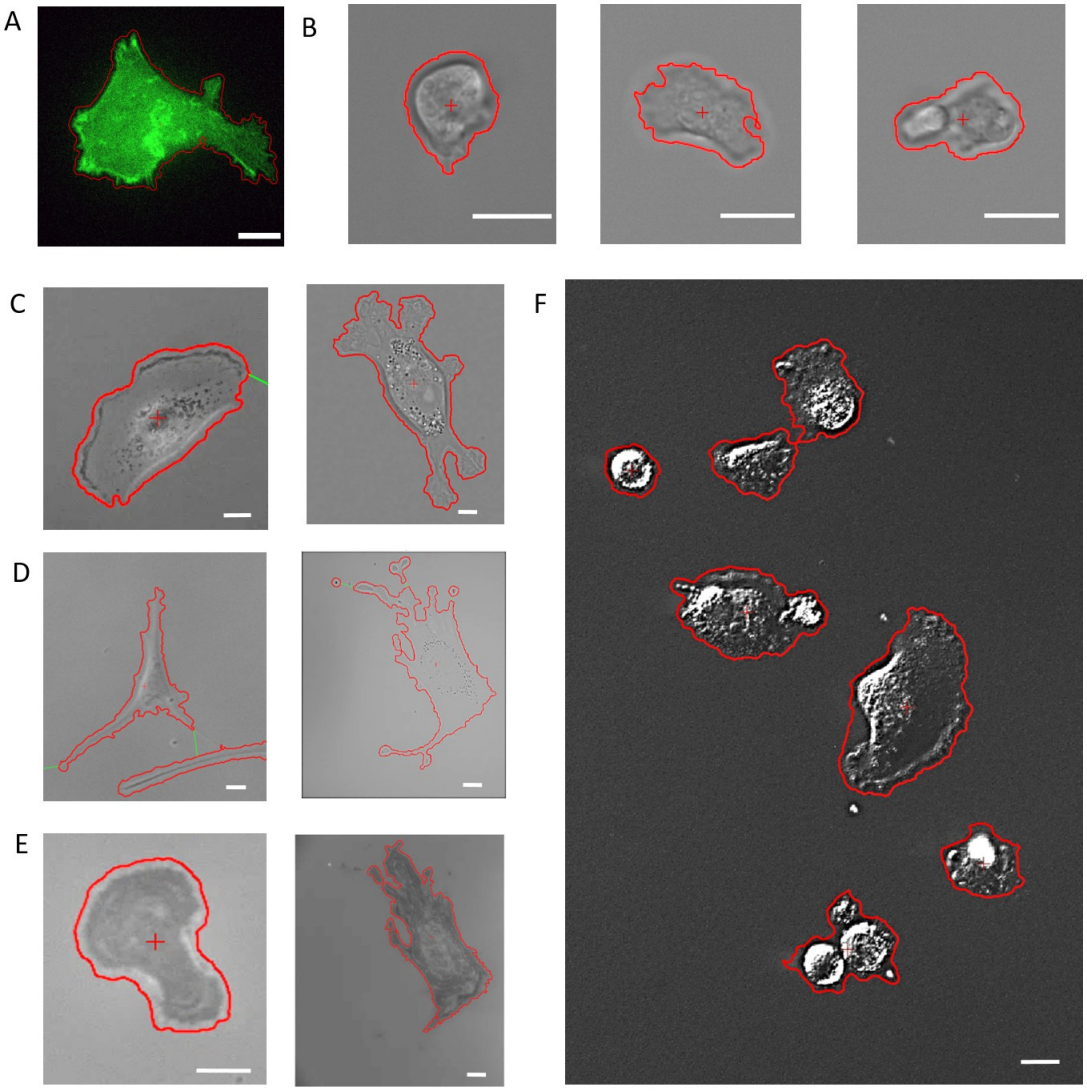
Robust segmentation via optical flow across optical modalities, cell types, and phenotypes. Scale bars are 10μm. All smoothing disks were set to 1 pixel and size filters 1000 pixels unless otherwise noted. A) A549 carcinoma cell stably transfected with GFP actin to serve as a baseline, optical flow threshold Th = 0.003, 0x magnification. B) Dictyostelium cells under 40x TL (Th = 0.01) exhibited a range of phenotypes: Amoeboid (left), keratocyte (center), and intermediate (right). C) MDA-MB-231 cells accurately segmented under 10x phase contrast (left, Th = 0.01) or 40x TL (right, Th = 0.03). D) Hs27 Fibroblasts accurately segmented under 10x phase (left, Th = 0.08) or 40x TL (right, Th = 0.0005). E) Both MDA-MB-231 cell (left, Th = 0.001) and Hs27 fibroblast (right, Th = 0.0005) segmented under IRM. F) Multiple MDA-MB-231 cells featuring a variety of morphologies accurately segmented under a single threshold value Th = 0.03 at 20x DIC. All images have been contrast enhanced for better visualization.

To validate the optical flow segmentation algorithm and demonstrate its robustness, segmentation of a variety of cell types and optical modalities are shown in Fig 3. To serve as a baseline for typical segmentation techniques, a fluorescently labeled A549 cell is easily segmented based on the cell’s motion between consecutive time frames rather than the fluorescent tag intensity in Fig 3A. Next, Dictyostelium cells are segmented without the use of any labels under transmitted light (TL) microscopy that is standard for most cell biology laboratories in Fig 3B. Dictyostelium was chosen to highlight the ability of optical flow to segment many different morphologies, behaviors, or otherwise phenotypes any given cell type exhibit, as cells of different phenotypes can exhibit drastically different morphologies and thus image characteristics [27, 28]. In Fig 3B, Dictyostelium cells exhibiting amoeboid, keratocyte/fan, or oscillator/intermediate phenotypes are segmented, each of which has a distinct morphology and migratory characteristics. Amoeboid phenotypes typically possess a rounded morphology leading to high contrast boundaries around their edge when imaged under TL or phase contrast microscopy, whereas keratocyte phenotypes exhibit a flatter, broad lamellipodia that typically have much lower contrast at the cell boundary comparatively. The intermediate phenotype switches between morphologies that look similar to both amoeboid and keratocyte, and all three phenotypes are segmented accurately via optical flow despite these differences in morphology and image characteristics.

Similarly, fibroblasts (Hs27) and epithelial (MDA-MB-231) cells were segmented under TL, phase contrast, differential interference contrast (DIC), and interference reflection microscopy (IRM). Phase, DIC, and TL are commonly used modalities in most cell biology labs, with cells imaged under TL having much lower contrast compared to phase or DIC typically. IRM is a lesser employed, but powerful modality. Fig 3C & 3D show both cell types under phase and TL modes, covering a range of complex morphologies between the two cell types. The optical flow algorithm is able to segment all cells tested regardless of morphology or optical modality due to the use of cell motion between consecutive images as a means of segmentation. Fig 3C shows two different typical morphologies of MDA-MB-231 cells exhibit—a spread out morphology in phase contrast and a more complicated, five lamellipodia morphology under transmitted light that are both accurately segmented via optical flow. Similarly, the optical flow algorithm is able to segment fibroblasts exhibiting multiple lamellipodia in complex geometries accurately under both phase and TL in Fig 3D. Even within the same field of view, the optical flow algorithm is able to segment cells of different morphology, as shown in Fig 3F where multiple MDA-MB-231 cells are segmented under 20x DIC, spanning clumped, balled up, spread and fan-like morphologies with the same flow threshold.

**Fig 4:**
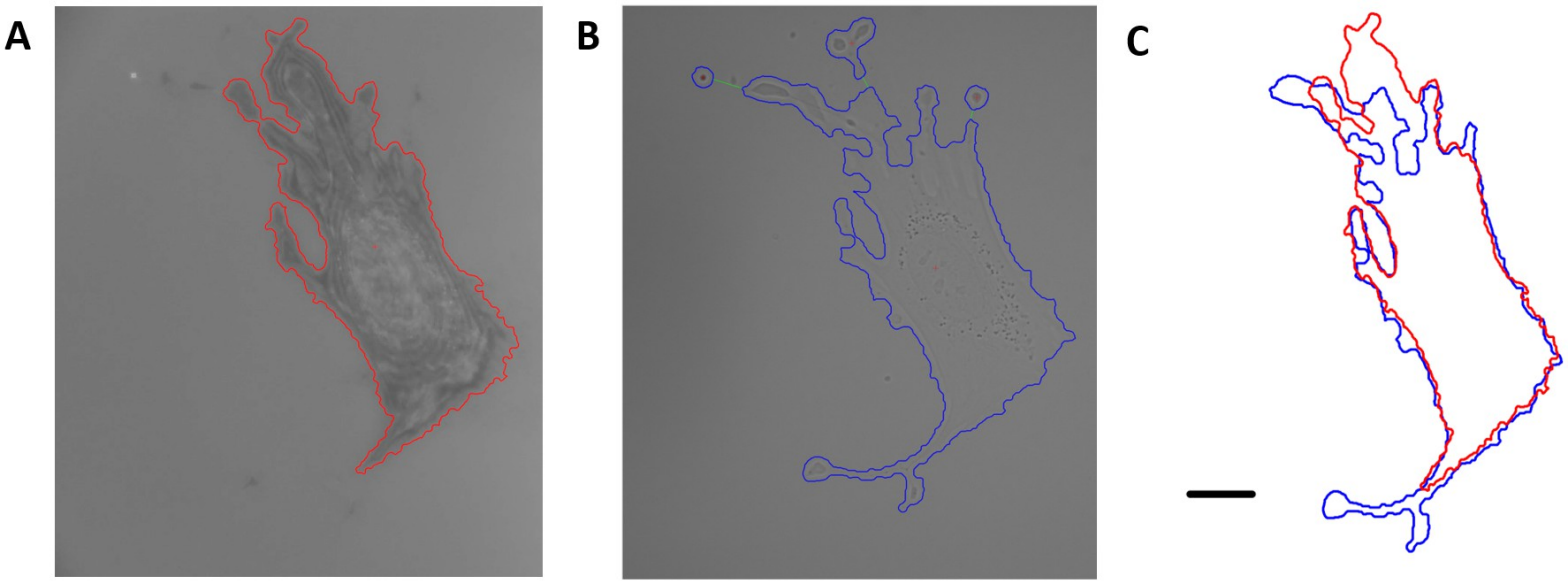
Direct comparison of a Hs27 fibroblast under A) 40x IRM and B) 40x TL. IRM highlights the cell-substrate interface while TL projects the entire 3 dimensional cell onto a 2 dimensional image, leading to fairly different segmented areas, compared in C). Scale bar is 20um.

IRM is a unique modality that visualizes interference patterns generated from reflections of an incident beam of light as it passes through materials of different refractive indices, highlighting the interactions at the cell-substrate interface [29]. In Fig 3E, both MDA-MB-231 and Hs27 cells are segmented accurately via optical flow despite having extremely different intensity or imagery characteristics compared to more traditional modes of microscopy. Indeed, IRM can reveal intricate cellular structures oftentimes not readily visible under transmitted light [30], and it is worthwhile to note that the multi-modality capability of the optical flow algorithm allows for direct comparison of observed cell behavior in the case of IRM vs TL. Fig 4 shows a direct comparison of the segmentation via optical flow of a fibroblast under both TL and IRM shown in Fig 3E, highlighting the different aspects of the fibroblast each modality is suited to detect. Such a comparison could be useful considering that it is common for cell adhesion to be characterized by measuring the area of spread cells, however it has been shown that the area observed under IRM is directly related to the degree and strength of cell adhesion [30].

A unique capability arises as a consequence of utilizing cell motion as a means for segmentation—the ability to distinguish cells in complex, yet stationary environmental surroundings. This is of particular interest as it is becoming increasingly common for research groups to observe cells interacting with various fabricated surfaces and structures as a means to elucidate underlying mechanisms governing cell behavior. *In-vitro* platforms investigate fundamental cellular processes such as adhesion and migration through a plethora of two or three-dimensional surfaces/structures including printed lines of ligands [31], nanodots [32], nanopillars [33], and 3D matrices [34]. Each one of these techniques results in a unique architecture present in images that pose a distinct challenge for segmenting cells interacting with these structures, often times resulting in the use of fluorescent or manual labeling to distinguish cellular boundaries from fabricated structures. One such example are platforms that investigate contact guidance, in which repeating grooves are etched or fabricated into a substrate to create three dimensional structures to investigate cellular response to topographical cues [35, 36].

**Fig 5:**
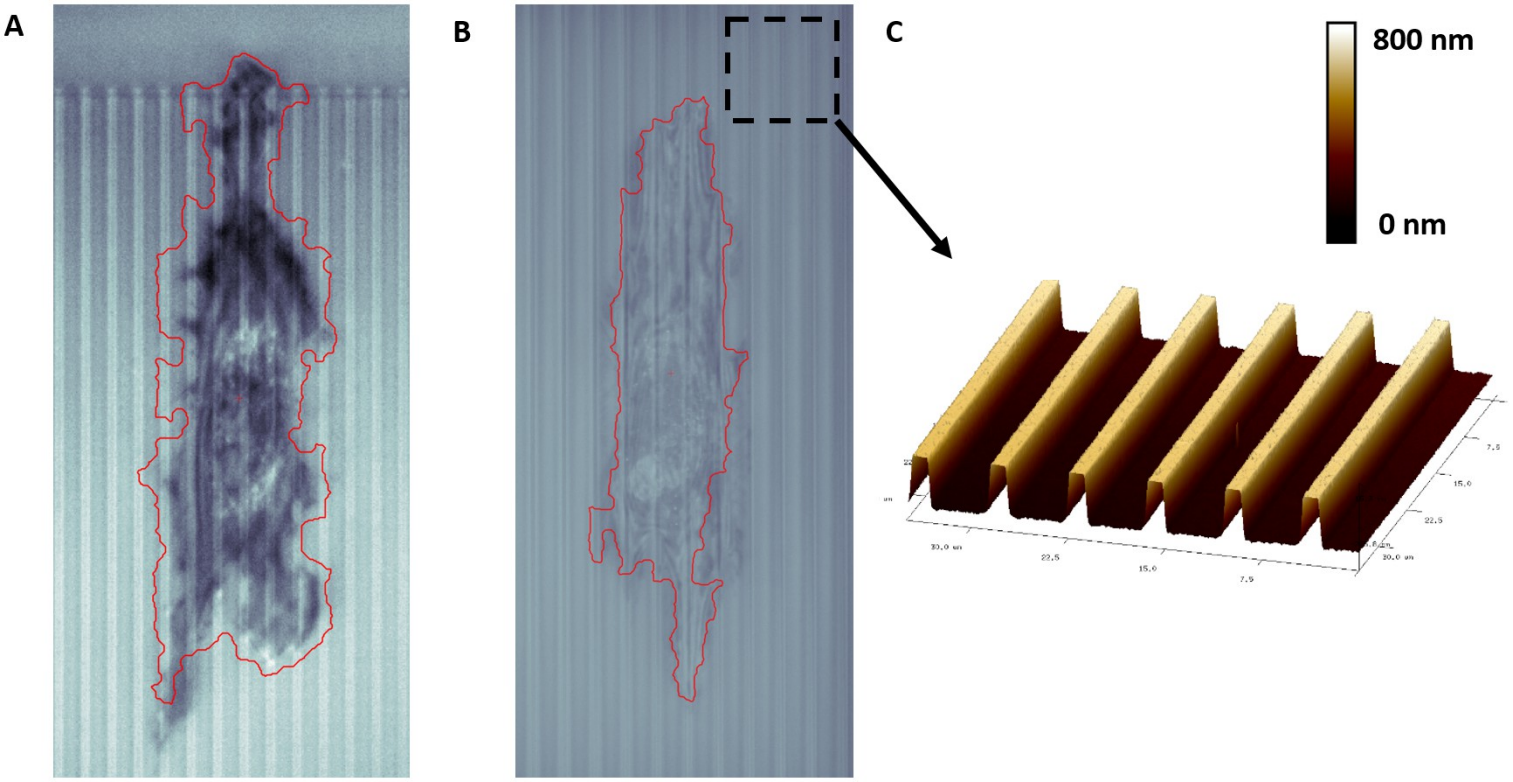
Hs27 under IRM with a 40x objective, atop etched contact guidance grooves of depth (D), ridge (R) and groove (G) dimensions A) D = 0.330 um, R = 2 um, G = 4 um, and B) D = 0.725 um, R = 2 um, G = 8 um. A flow threshold of Th = 0.01 (A) or 0.02 (B) is able to segment the fibroblast with reasonable accuracy under diverse *in-vitro* imagery. C) Atomic Force Microscope image depicting three-dimensional view of the contact guidance grooves.

Our group recently introduced a contact guidance platform capable of integrating with nearly all forms of live-cell microscopy [37], which generates a unique problem for accurate cell segmentation against the backdrop of etched grooves that serve as topographical cues. Fig 5 shows Hs27 fibroblasts atop such grooves under IRM, in which the etched grooves can scatter/diffract light differently depending on their topographical dimensions, leading to imagery data much more complex than a traditional flat substrate or petri dish. The issue is further complicated by the fact that the relative intensity/pixel values of the topographical structures can be quite similar to those of a cell, making accurate segmentation exceptionally challenging. However, due to the stochastic fluctuations of cell motion, our optical flow algorithm segments the cell with reasonable accuracy against the backdrop of the etched structures with a threshold value of Th = 0.01–0.02. To the best of our knowledge, the presented optical flow algorithm is the only option for label-free segmentation of cells interacting with such structures in a highly generalizable manner.

### Evaluation

To evaluate segmentation by optical flow versus commonly used segmentation approaches, we assembled a data set consisting of various cell types and magnifications spanning the optical modalities used for optical flow validation (phase, DIC, TL, IRM, and fluorescence in S1–S5 Figs in S1 File). Two commonly used metrics to evaluate each method were calculated: F-score and Jaccard index for method detection accuracy and segmentation accuracy, respectively [10]. In each image, cells are manually segmented to serve as ground truth and compared against the segmentation mask produced by each method. The true positives (TP), false positives (FP), and false negatives (FN) of each method under each optical modality are calculated, and the F-score metric defined below:

$$F_{1}=\frac{TP}{TP+\frac{1}{2}(FN+FP)}$$ (3)

Along similar lines, for each data set containing the ground truth of cells *T*, the Jaccard index for each method that segments cells *S* is given by:

$$J\left(T,\;S\right)=\frac{\left|T\cap S\right|}{\left|T\cup S\right|}$$ (4)

As live-cell microscopy has advanced to collect large data sets using a range of optical modalities, the onus is on segmentation methods that are both quick to optimize and robust across different data sets within an experiment. This is particularly true as systems are increasingly capable of multiplexing, or exploring many different experimental conditions that increases throughput but can cause changes in imagery within a single experiment (i.e. sidewall effects, illumination gradients, etc.) [38]. Thus, a brief description of the level of training and the labeled data vs output for Ilastik and FastER are shown in the S1 File (See S7 and S8 Figs in S1 File). The approximate time it took to optimize these segmented images (*not* computation time) is also tabulated for each method and data set.

The *F*_*1*_ scores, Jaccard index, and optimization times are summarized in Table 1. Two popular trainable machine learning platforms that have a user-friendly interface, Ilastik [39] and fastER [10], were chosen for comparison to optical flow. The highest rankings/lowest optimization times for each data set are highlighted in bold. FastER had the lowest rankings for these conditions, requiring extensive training time per optical modality, and simply did not have enough data to train off of for the higher magnification, single cell data sets. Ilastik, another powerful machine learning platform, performed much better under these conditions but still required extensive iterative training, and thus took more time before satisfactory segmentation was achieved. By comparison, the optical flow segmentation took a small fraction of the time of the other two methods utilized, while typically having higher or approximately equal *F*_*1*_ scores and Jaccard indices in the various optical modalities of this data set. It should be noted that this comparison is not meant to be exhaustive as it is inherently subjective to judge when segmentation is satisfactory, but rather convey the amount of manual labor often “hidden” in the requirements of most readily available methods compared to the optical flow method outlined here.

**Table 1:** Summary of evaluation rankings of F_1_ scores, Jaccard indices, and optimization time of optical flow and contemporary machine learning methods.

Data set	Method	F_1_ score	Jaccard Index	Time (min)
10x phase	Optical Flow	**0.73**	**0.59**	**2**
	Ilastik	0.68	0.52	15
	FastER	0.49	0.32	15
20x DIC	Optical Flow	**0.90**	**0.82**	**2**
	Ilastik	0.88	0.79	15
	FastER	0.62	0.45	15
40x TL	Optical Flow	0.88	0.77	**2**
	Ilastik	0.88	**0.79**	10
	FastER	n/a	n/a	n/a
40x IRM	Optical Flow	**0.93**	**0.87**	**1**
	Ilastik	0.91	0.84	5
	FastER	n/a	n/a	n/a
40x Fluorescence	Optical Flow	0.94	0.88	**1**
	Ilastik	0.94	**0.89**	5
	FastER	n/a	n/a	n/a

## Discussion

Here we have introduced an optical flow based strategy for label-free segmentation of cells. By utilizing the changes in image intensity (*I)* as a function of position (x, y) and time (t) as a means to differentiate areas that belong to cells versus the background, the burden of segmentation is largely removed from cell intensity characteristics (i.e. contrast) and shifted towards how that intensity changes from image-to-image. This is a novel way of interpreting live-cell imagery, and one can think of this as additional information that exists for every pixel in a typical image histogram, but has been seemingly overlooked. This approach has three clear advantages to contemporary segmentation techniques, which typically require extensive parameter-based optimization or require manual labeling of training data. First, the proposed optical flow method is relatively simple to use–reducing the amount of parameters required for tuning down to two, making it time effective to process multiple data sets in comparison to contemporary techniques (see Table 1). Second, this optical flow method is capable of accurate segmentation without any specificity or otherwise training with regards to optical modality, cell type, or experimental platform (see Fig 3). Third, segmentation results from our approach are readily interpretable since they can be traced directly back to the physics of motion. In contrast, with deep learning techniques that automatically generate thousands of parameters, as well as with techniques which incorporate dozens of tuning parameters, it can be difficult to determine why the software did or did not give adequate results. These three advantages result in a robust technique that is both accessible to researchers across laboratories (see S1 File) and capable of time-effective, high throughput image analysis.

The use of the term cell “movement” that is used as a means of cell segmentation is perhaps misleading, as it infers a drastic (i.e. noticeable) translation of the cell position between time frames. However, in our experience, even cells that appear stationary between time frames exhibit significant optical flow within the boundary of their area due to intensity fluctuations from intracellular processes that are readily segmented, usually with the same flow threshold Th suitable for segmentation of motile cells within the same experimental/optical/imagery conditions. These benefits combined with relatively few and simple parameters that require tuning make optical flow segmentation strategies appealing to the broader cell biology community.

Optical flow based strategies outlined here do however have some shortcomings and limitations. First, the accurate segmentation under optical flow requires a stable experimental set up such that the only movement between consecutive frames is that of the cells and not the experimental background itself. As such, drift of the microscope stage or objective focus can lead to a large displacement of pixel intensity values, and thus large values of optical flow that are unrelated to actual cell motion. To combat this, image alignment software and autofocus methods are recommended except at the lowest magnifications. On top of the experimental set up, the frequency of data collection is an important factor that can directly affect the quality of segmentation under optical flow. Two of the underlying assumptions of the optical flow algorithm is that the net intensity is conserved and that the net displacement is small between consecutive frames. Thus, the longer time elapsed between frames the more likely objects (i.e. cells) move greater distances, and the less likely these assumptions are to be valid. In our experience with the variety of cells/experiments outlined in this manuscript, time steps of 10 min yielded similarly accurate cell segmentation as time steps of 20s, however this is likely to be highly dependent upon the cell type and experimental conditions. Last, similar to many other label-free segmentation approaches, is there is no inherent way to distinguish if a segmented object is a single cell or a multicellular aggregate (i.e. Fig 3F). The issue of segmenting cells in physical contact, or declumping, is a technical challenge that has garnered much attention towards solving ranging from image processing techniques [8, 9] to machine learning approaches [40]. Currently, the proposed optical flow algorithm is suitable for single cell segmentation (i.e. migration or adhesion characterization). Future work could incorporate declumping methods, such as application of watershed transform or intensity thresholding within segmented objects to unmerge conjoined segmented cells. Furthermore, as the amount of manual intervention required is minimal (two intuitive parameters), this optical flow based method is an appealing strategy to build upon for future work on completely automated cell segmentation techniques.

## Conclusion

The algorithm presented here, to the best of our knowledge, is the first optical-flow based label-free technique that offers relatively simple and robust means of cell segmentation. The notion of utilizing cell motion as a means to distinguish cells from their background is a rather elegant strategy for segmentation in a variety of environments or optical modalities, without the need for labels. It is worth noting that the use of optical-flow based segmentation is not exclusive to other image processing techniques, opening the possibility of optical-flow to be combined with segmentation techniques such as machine learning, for potential improvements in segmentation accuracy, robustness and ultimately classification. With robust segmentation capabilities and few parameters to manually optimize, the ease-of-use of this optical flow segmentation algorithm stands to offer accessibility into the dynamic behavior of cells for typical cell biology laboratories across disciplines.

## Materials

### Cell cultures

All human cell lines were purchased through ATCC (Hs27 #CRL-1634, MDA-MB-231 #HTB-26, A549 #CCL-185), and cultured according to ATCC protocols in DMEM (ATCC #30–2002) in 10% fetal bovine serum (ATCC #30–2020) at 37°C in 5% CO_2_. Cells were subcultured according to ATCC protocols and cells were harvested between 30–80% confluence for all experiments. A549 cells were transfected for the stable expression of GFP-actin (LifeAct) as previously described [33]. Wild-type *Dictyostelium discoideum* cells of the AX2 strain generously obtained from the Devreotes laboratory (Johns Hopkins University, USA) were used in this study and were cultured axenically in HL5 media at 22°C as outlined in [27]. For experiments involving *Dictyostelium* imaging, cells were harvested at ~80% confluence by gently aspirating/rinsing the culture dish/flask and using the supernatant of suspended cells for live cell imaging.

### Microscope/in vitro set ups

All live cell experiments were conducted on Ziess Z1 Axio Observer microscope and imagery collected on either a Hamamatsu ORCA R2, Hamamatsu ORCA Flash 4.0, or a Zeiss Axiocam 702 mono camera. Live cell imaging was performed using phase contrast (10X, 0.9 NA objective), Differential Interference Contrast (DIC, 20X, 0.8 NA objective) transmitted light (TL 40X, 1.4 NA objective), fluorescence (100X, 1.46 NA objective) or interference reflection microscopy (IRM) (40X, 1.4 NA objective) and collected on Zeiss Zen software. For mammalian cell lines: a heated stage and temperature controlled enclosure held the stage temperature at 37.0 ± 0.04°C (Zeiss) with humidity and CO2 regulated at 98% and 5%, respectively, by flowing a gas-air mixture through a heated water bottle and into the enclosure. For *Dictyostelium* imaging, cells were imaged in glass-bottomed petri dishes at room temperature (22°C). Focus was stabilized for the multi-hour long experiments using an integrated hardware-based focus correction device (Zeiss Definite Focus). All mammalian cell line experiments were done in serum free media, and conducted on either glass-bottomed well plates/petri dishes or in the case of contact guidance structures, quartz chips detailed previously [37]. The *Dictyostelium* imaging was conducted on glass bottomed petri dishes in HL5 culture media.

## Supporting information

S1 File Supporting figures and information for the manuscript. (DOCX)

S2 File Raw data zip. All the raw imagery/data used in this manuscript.
(ZIP)

S3 File Segmentation code. MATLAB file containing the optical flow algorithm outlined in this manuscript.
(M) Click here for additional data file.

S4 File Supporting MATLAB file 1. Supporting .m file needed to run segmentation code. Please see comments of segmentation code for use.
(M) Click here for additional data file.

S5 File Supporting MATLAB file 2. Supporting .m file needed to run segmentation code. Please see comments of segmentation code for use.
(M) Click here for additional data file.
