# Supplementary material for: Robust optical flow algorithm for general single cell segmentation
Source: PLoS One. 2022 Jan 14;17(1):e0261763. doi: 10.1371/journal.pone.0261763 (PMC8759635; doi:10.1371/journal.pone.0261763)
Supplement: S1 File — (DOCX) [file pone.0261763.s001.docx]

Robust Optical Flow Algorithm for General Single Cell Segmentation: Supplementary Information

Michael C. Robitaille^1^, Jeff M. Byers^1^, Joseph A. Christodoulides^1^, Marc P. Raphael*^1^

^1^ Materials Science and Technology Division, U.S. Naval Research Laboratory, Washington D.C.

* Corresponding author: Marc.Raphael@nrl.navy.mil

1. **Motivation**

**
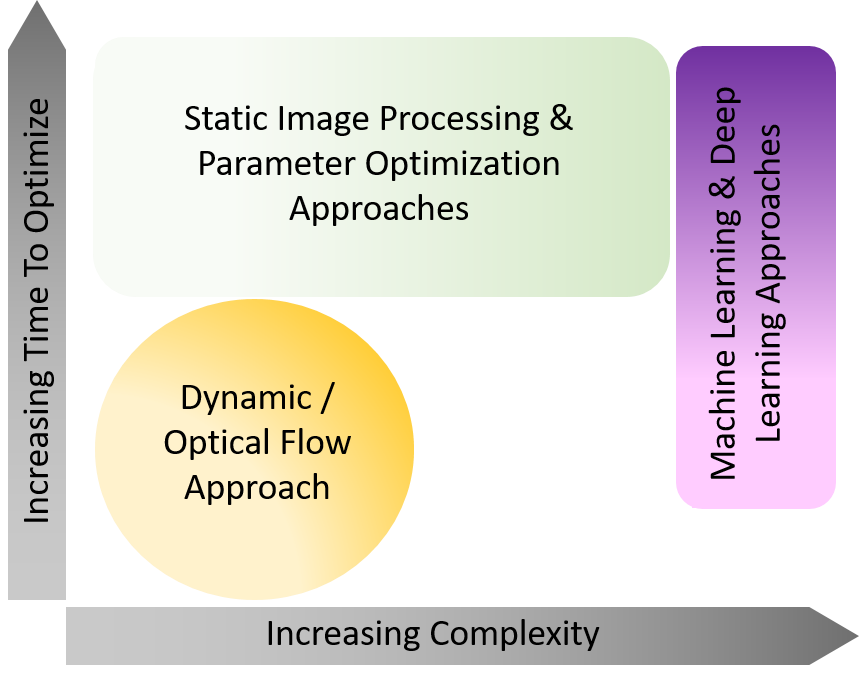
**

Figure S1: Phase diagram comparing the proposed optical flow approach to contemporary image processing and machine/deep learning techniques.

Image processing techniques offer a powerful way to distinguish cells from their background, such as CellProfiler(1). However, since the image processing steps depend directly upon the image characteristics, they often have to be re-optimized as those image characteristics change (e.g. change in cell type, optical modality, etc.). This results in long times to optimize segmentation for new experimental runs as well as increased complexity to the processing pipeline. Conversely, machine/deep learning approaches can offer shorter optimization times if the training library contains enough manually labeled data that is similar to the set of imagery to be analyzed. However, if the imagery is sufficiently different from the training library, new training data must be manually labeled and reapplied until satisfactory results are achieved, leading again to increased optimization times. Due to the opaque nature of some machine learning, and especially deep learning algorithms, the reasons behind the success or failure of these methods are often obscured. Conversely, by leveraging relative movement between consecutive frames in time-lapse imagery, the proposed optical flow approach offers robust segmentation across cell types/optical modalities with relatively few parameters to optimize that are intuitively easy to grasp. This leads to a simple and relatively fast way to segment diverse single cell imagery.

1. **Evaluation Data Set**

To evaluate the segmentation via optical flow to contemporary available segmentation techniques, two consecutive images of various cells at various magnifications were selected from each optical modality used in validation: fluorescence, phase contrast, differential interference contrast (DIC), interference reflection microscopy (IRM), and transmitted light (TL). An image from each data set is show in figures S2-S6. The training or parameter optimization and resulting masks/overlays are shown in figures S7-S9 for the phase contrast data set, and a direct comparison is shown in figure S10.


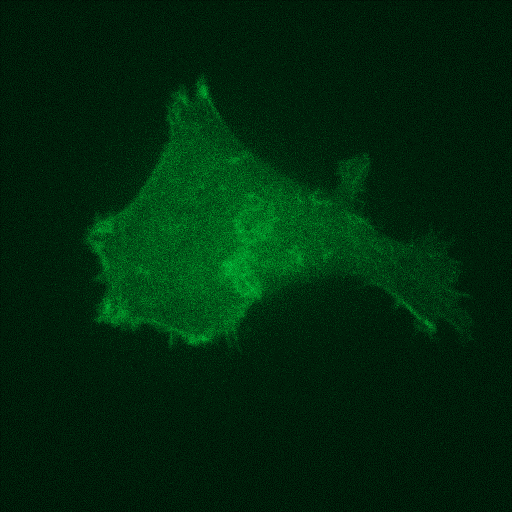


Figure S2: Fluorescence data set of a single A549 cell under 100x magnification, time step of 10s. Scale bar 10 um.


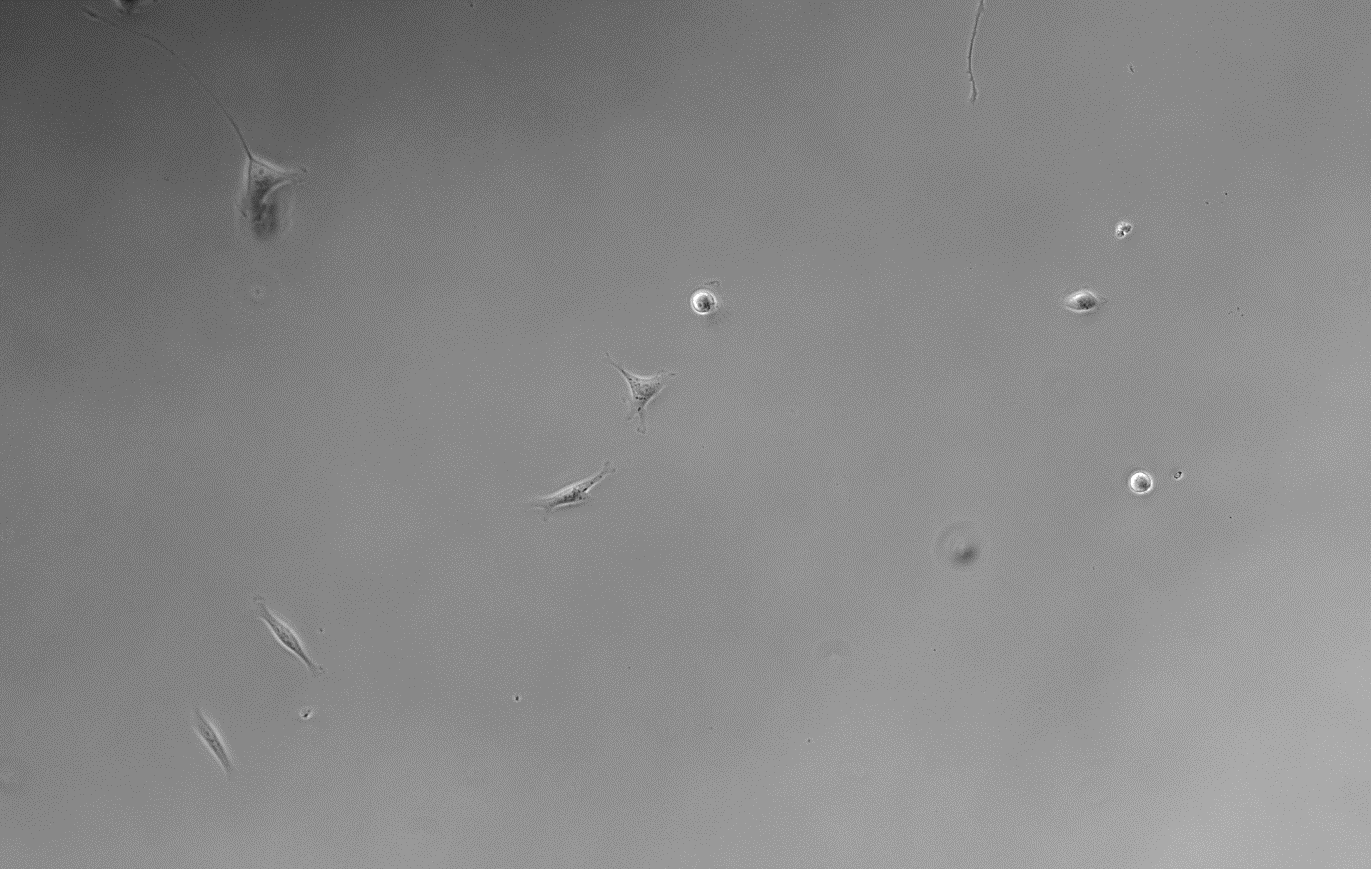


Figure S3: phase contrast data set of a MDA-MB-231 cells under 10x magnification, time step of 600s. Scale bar 40 um.





Figure S4: DIC data set of MDA-MB-231 cells under 20x magnification, time step of 300s. Scale bar 20 um.


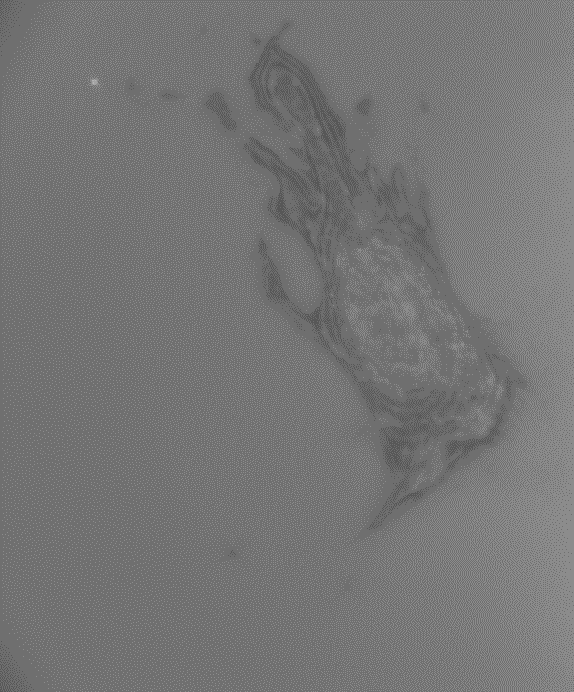


Figure S5: IRM data set of a single Hs27 fibroblast cell under 40x magnification, time step of 600s. Scale bar 20 um.


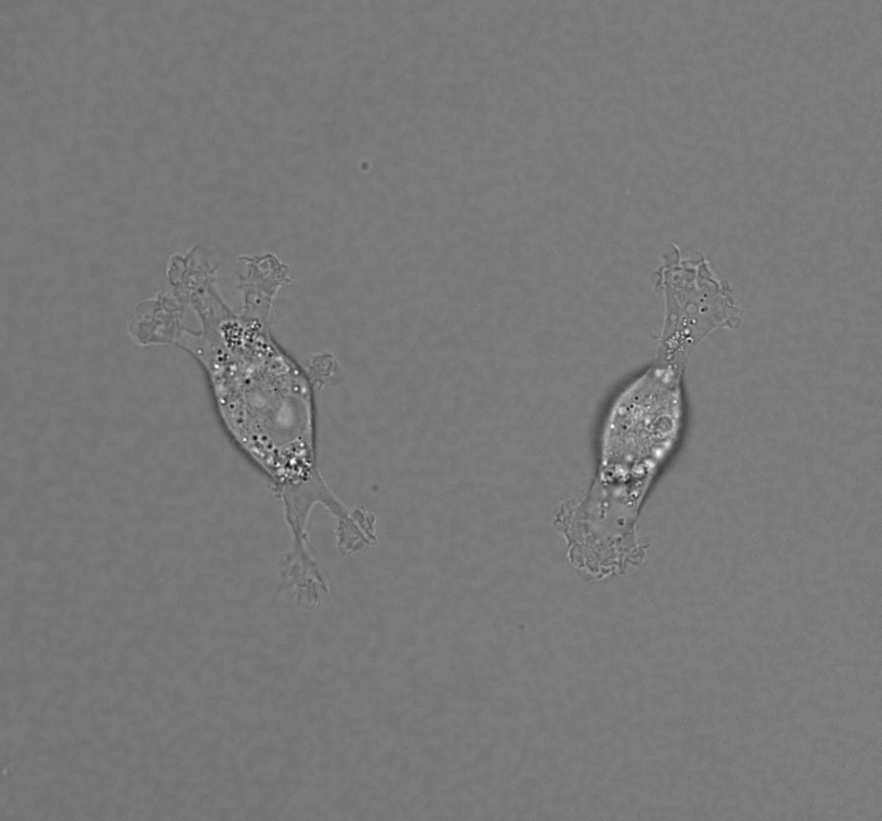


Figure S6: TL data set of two MDA-MB-231 cells under 40x magnification, time step of 300s. Scale bar 20 um.

1. **FastER (phase contrast)**


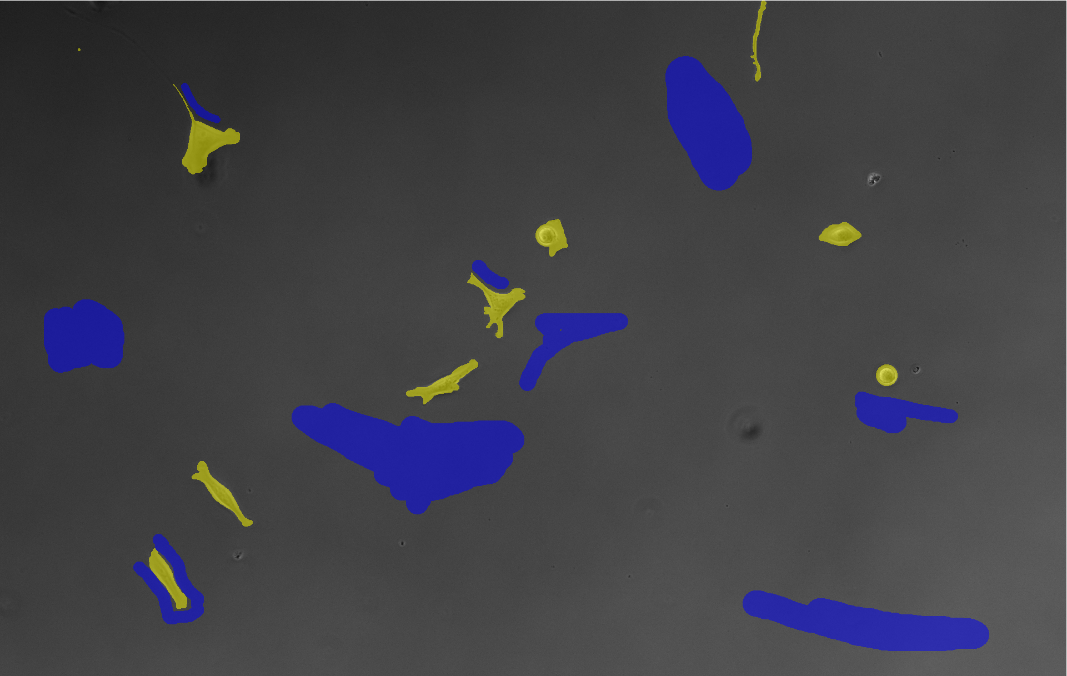

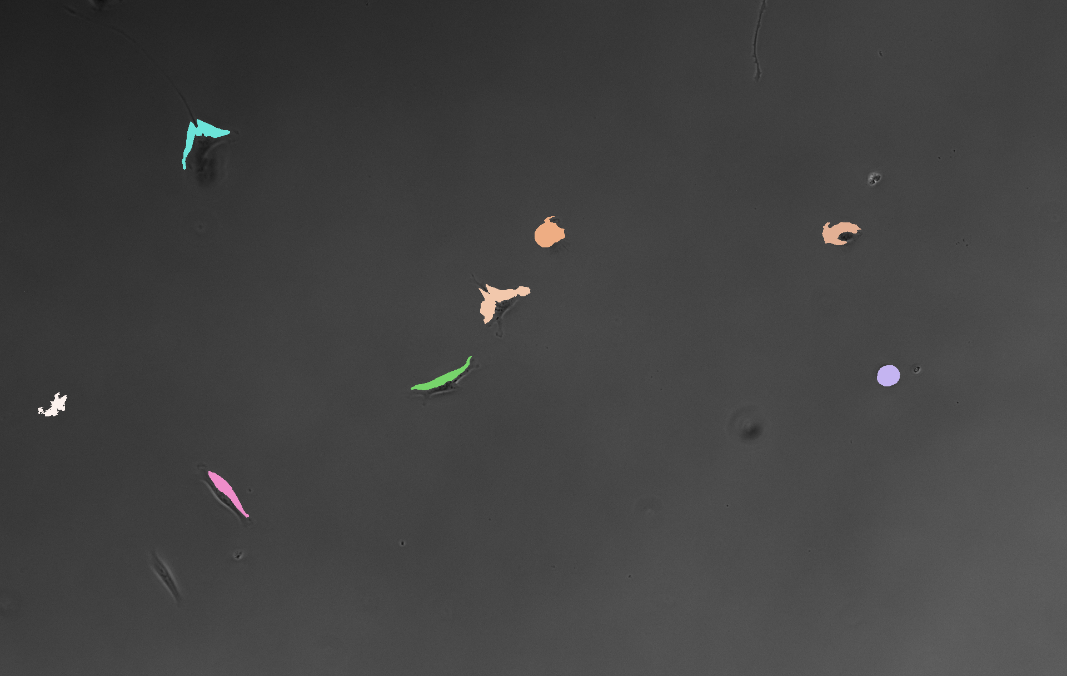


Figure S7: (Top) Manually labeled over 10 cells (yellow) and 10 background (blue) areas over two images and (Bottom) the resulting segmentation via FastER. Optimization time approximately 15 minutes

**Ilastik**

…

1. **Ilastik (phase contrast)**


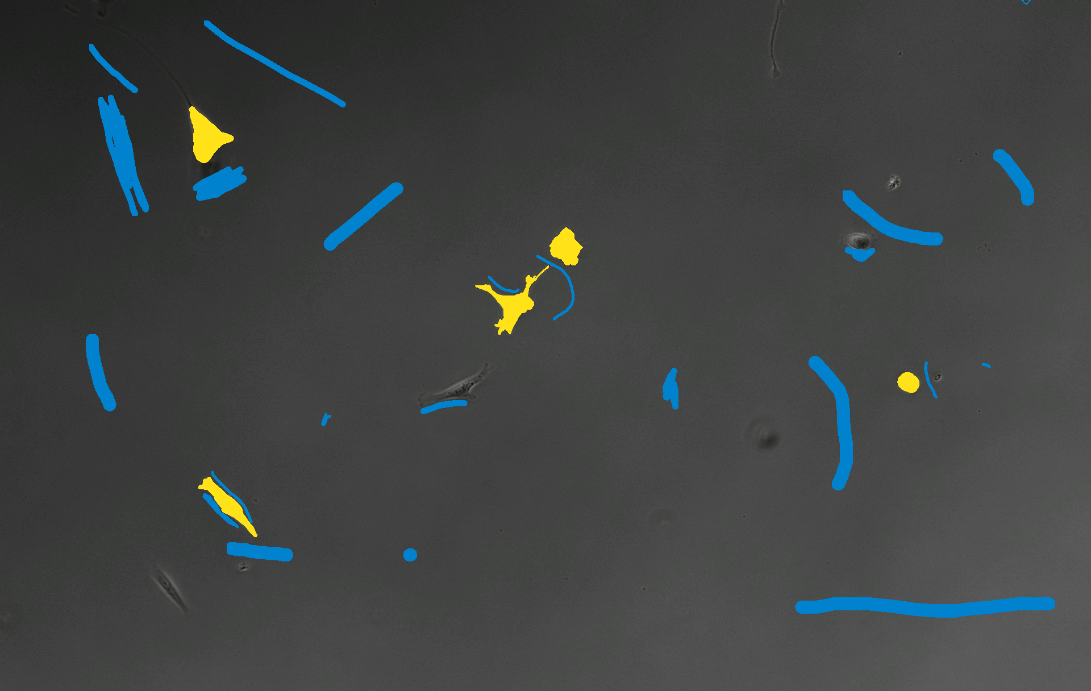

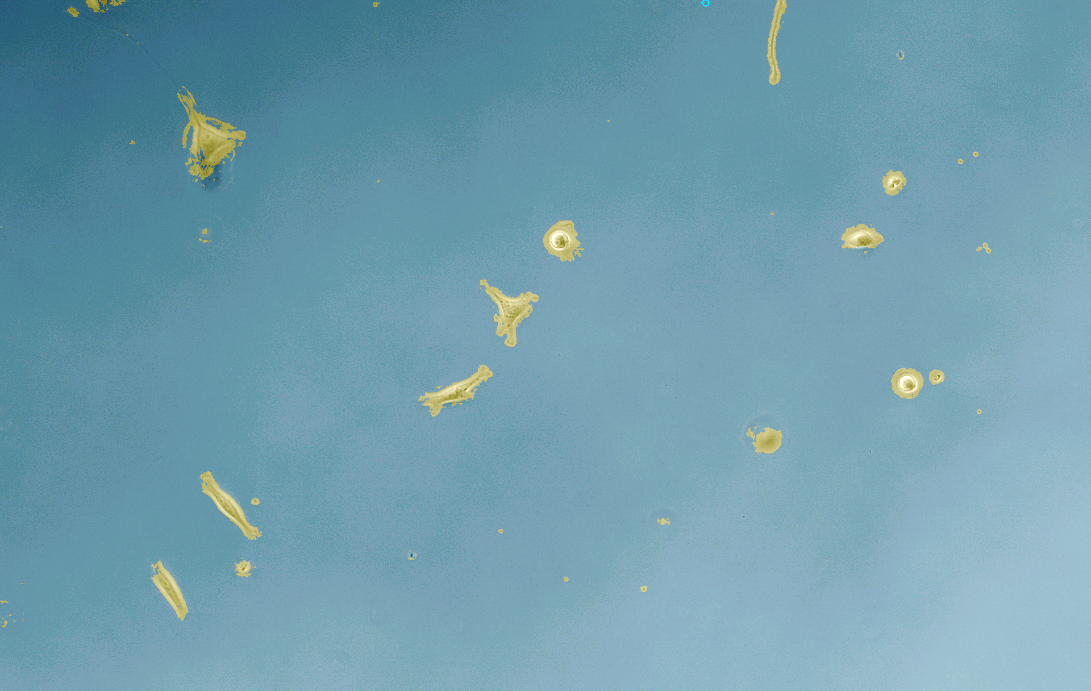


Figure S8: (Top) manually labeled over 5 cells (yellow) and over 20 background areas (blue) in iterative training, and (Bottom) the resulting segmentation via Ilastik. Optimization time 15 minutes.

1. **Optical Flow (phase contrast)**


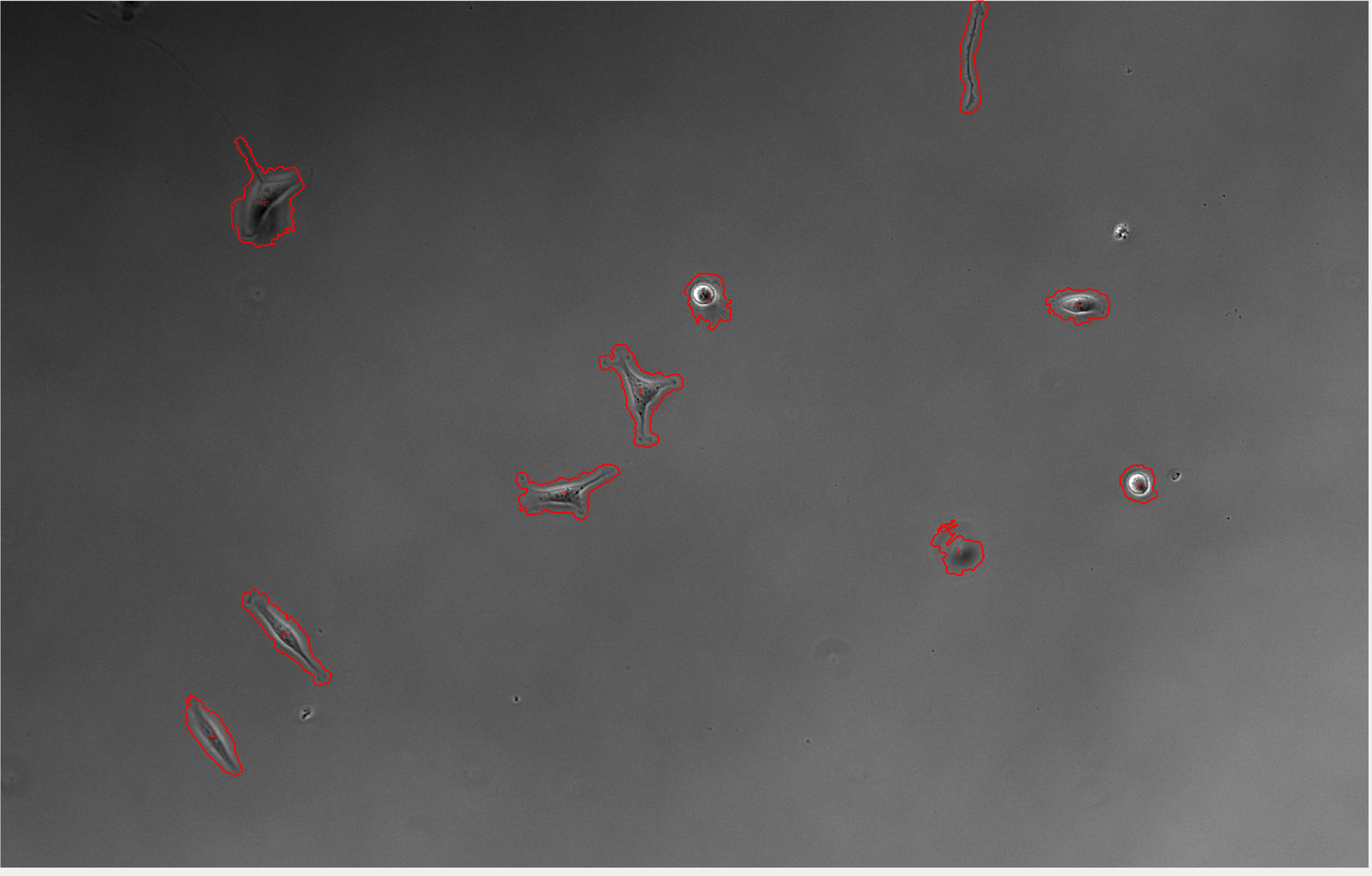


Figure S9: manually adjusted 2 parameters (th = 0.05, disk = 1), optimization time approximately 2 minutes

1. **Method Comparison**


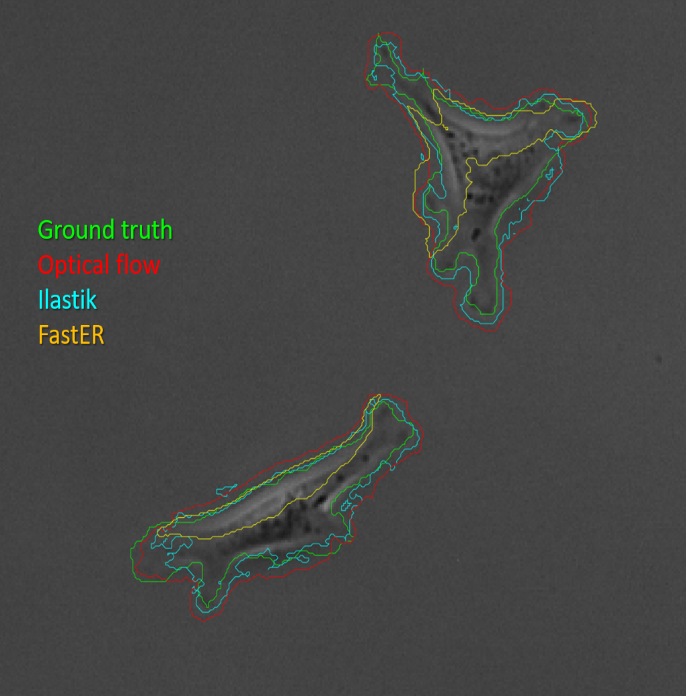


Figure S10: Segmentation overlay on MDA-MB-231 cells under phase contrast cell for method comparison.

**REFERENCES**

1. Carpenter AE, Jones TR, Lamprecht MR, Clarke C, Kang IH, Friman O, et al. CellProfiler: image analysis software for identifying and quantifying cell phenotypes. Genome biology. 2006;7(10):R100.
