# Supplementary figures and images for: Robust optical flow algorithm for general single cell segmentation
Source: PLoS One. 2022 Jan 14;17(1):e0261763. doi: 10.1371/journal.pone.0261763 (PMC8759635; doi:10.1371/journal.pone.0261763)

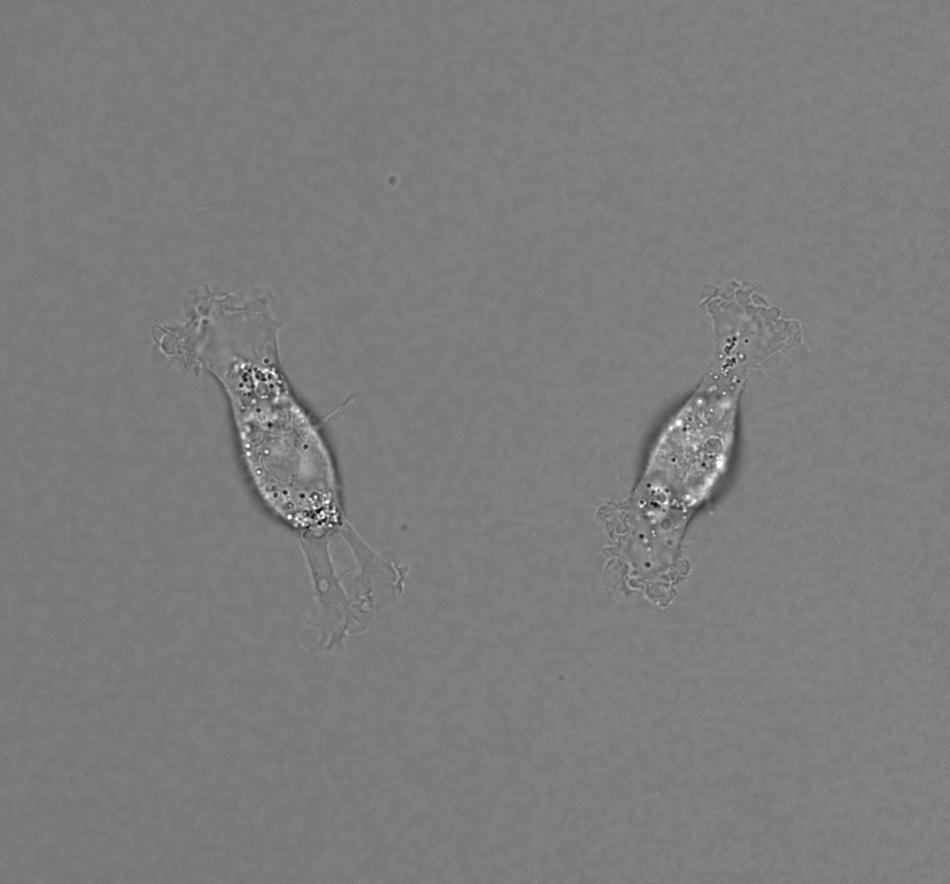

Supplement: S2 File — All the raw imagery/data used in this manuscript. (ZIP) [file pone.0261763.s002.zip › Raw Data/Figure 1 Imagery/Fig1_t01.tif]

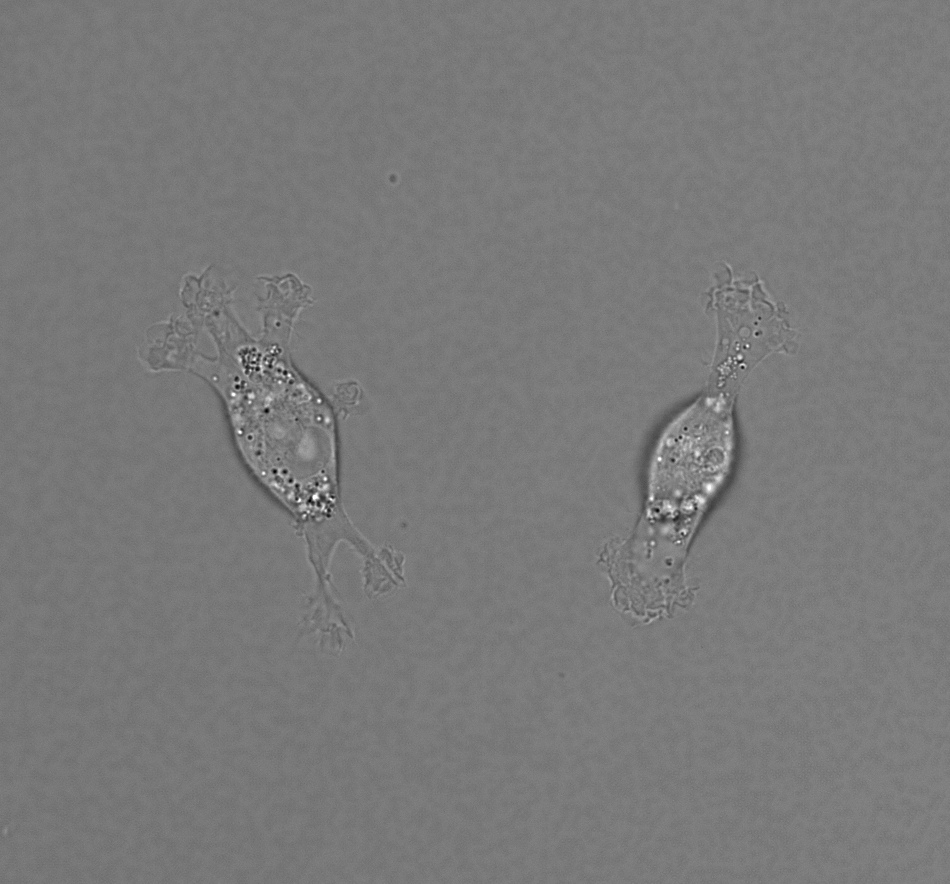

Supplement: S2 File — All the raw imagery/data used in this manuscript. (ZIP) [file pone.0261763.s002.zip › Raw Data/Figure 1 Imagery/Fig1_t02.tif]

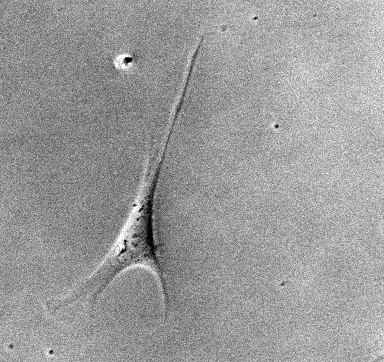

Supplement: S2 File — All the raw imagery/data used in this manuscript. (ZIP) [file pone.0261763.s002.zip › Raw Data/Figure 2 Imagery/a-b/Fig2a-b_t01.tif]

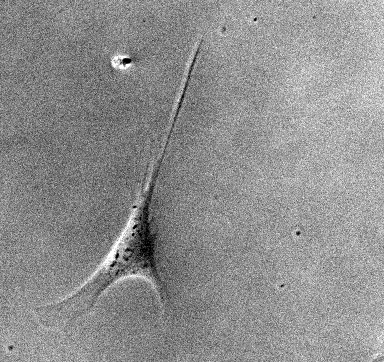

Supplement: S2 File — All the raw imagery/data used in this manuscript. (ZIP) [file pone.0261763.s002.zip › Raw Data/Figure 2 Imagery/a-b/Fig2a-b_t02.tif]

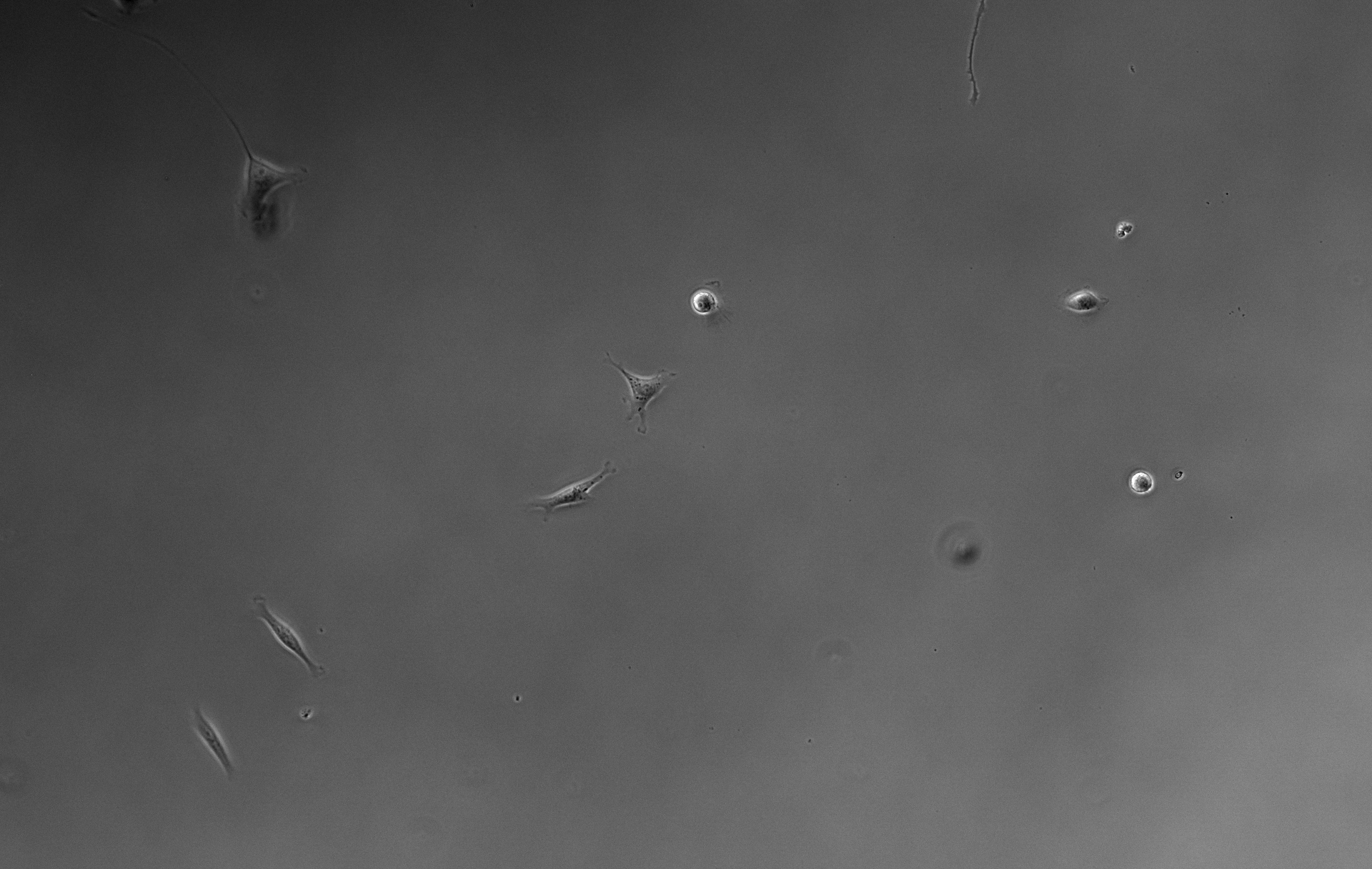

Supplement: S2 File — All the raw imagery/data used in this manuscript. (ZIP) [file pone.0261763.s002.zip › Raw Data/Figure 2 Imagery/c/Fig2c_t01.tif]

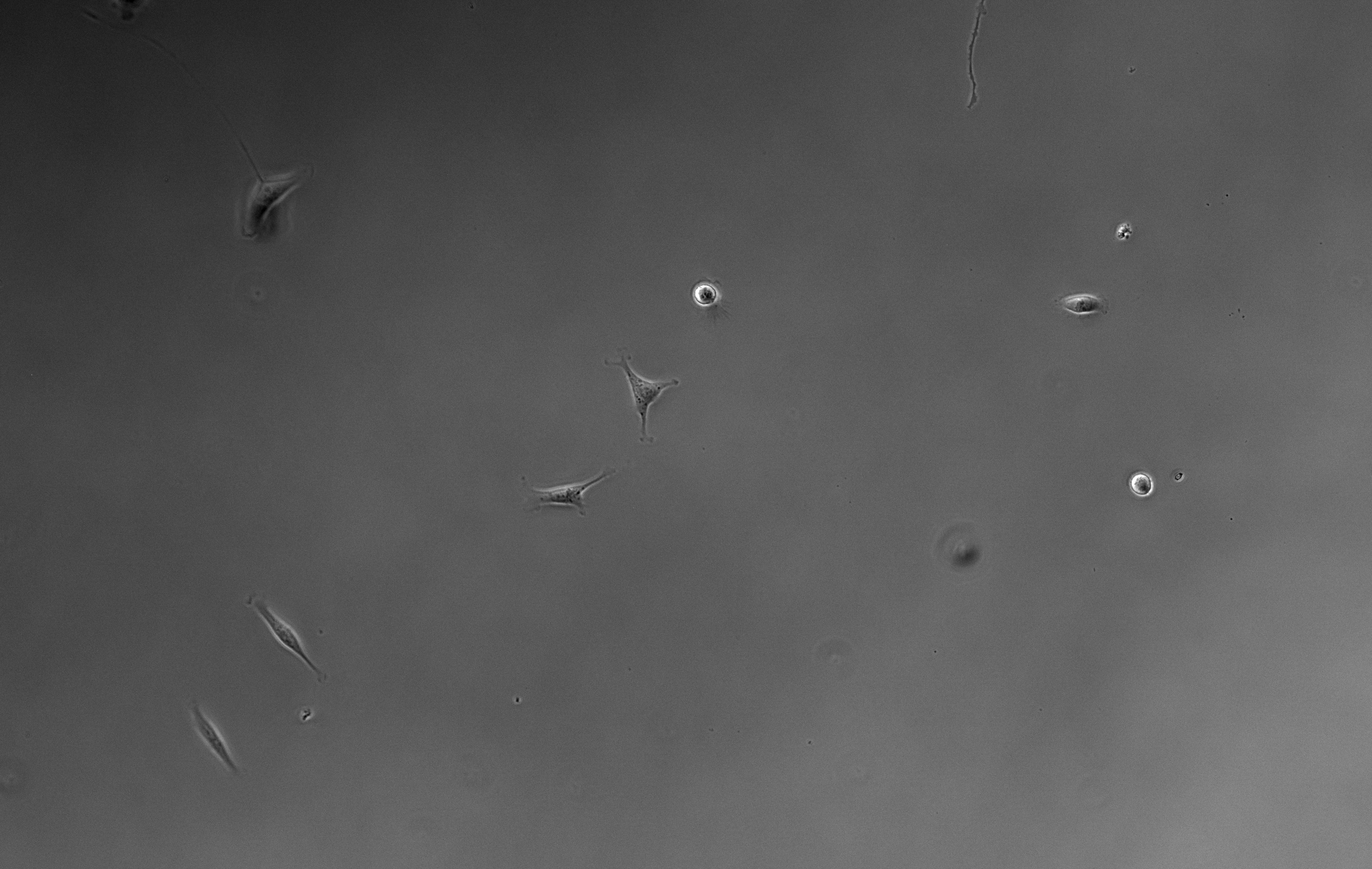

Supplement: S2 File — All the raw imagery/data used in this manuscript. (ZIP) [file pone.0261763.s002.zip › Raw Data/Figure 2 Imagery/c/Fig2c_t02.tif]

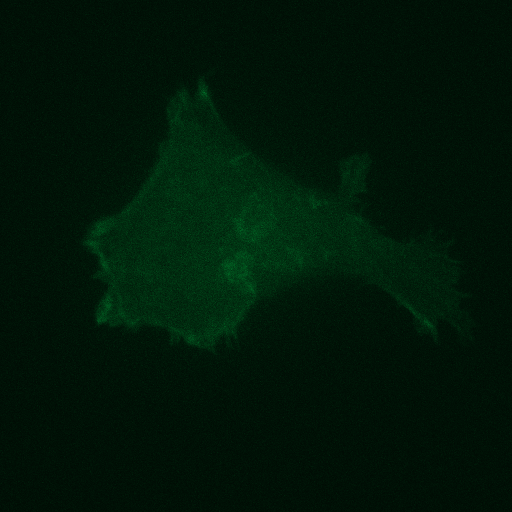

Supplement: S2 File — All the raw imagery/data used in this manuscript. (ZIP) [file pone.0261763.s002.zip › Raw Data/Figure 3 Imagery/a/Fig3a_t01.tif]

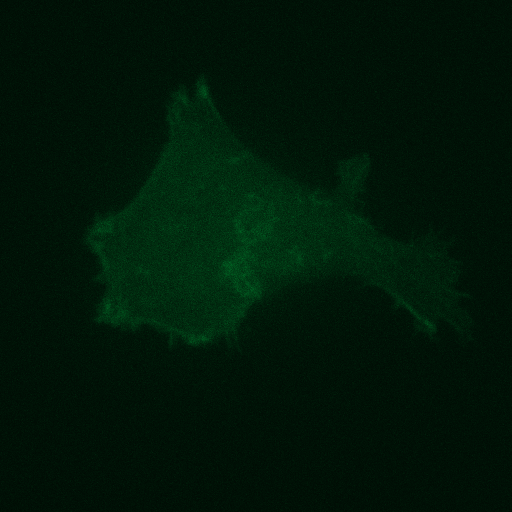

Supplement: S2 File — All the raw imagery/data used in this manuscript. (ZIP) [file pone.0261763.s002.zip › Raw Data/Figure 3 Imagery/a/Fig3a_t02.tif]

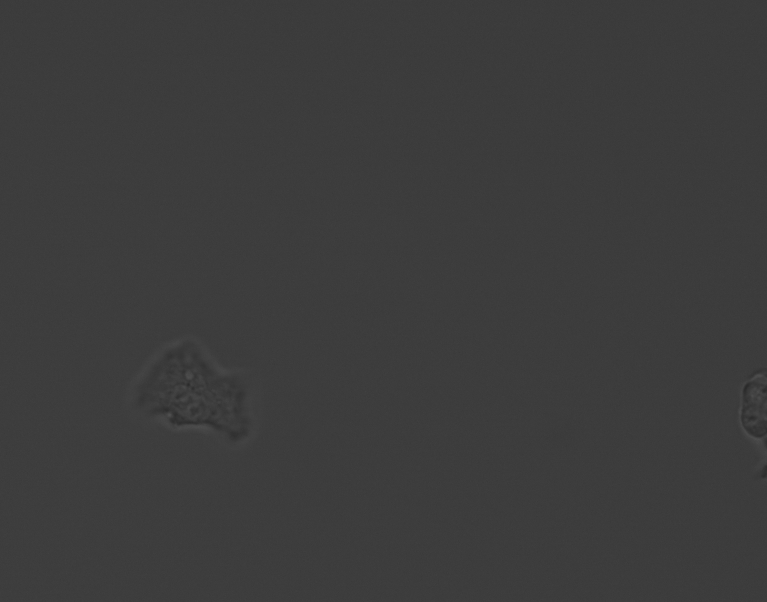

Supplement: S2 File — All the raw imagery/data used in this manuscript. (ZIP) [file pone.0261763.s002.zip › Raw Data/Figure 3 Imagery/b-center/Fig3b-1_t01.jpg]

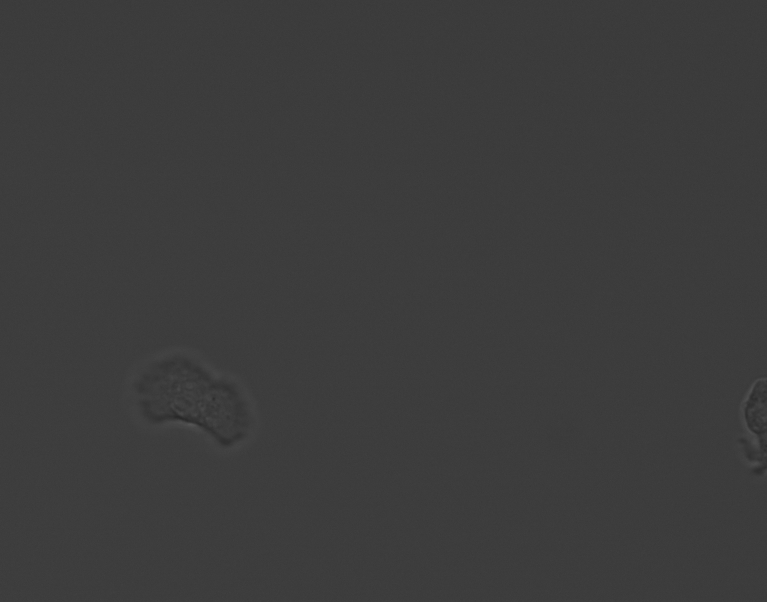

Supplement: S2 File — All the raw imagery/data used in this manuscript. (ZIP) [file pone.0261763.s002.zip › Raw Data/Figure 3 Imagery/b-center/Fig3b-1_t02.jpg]

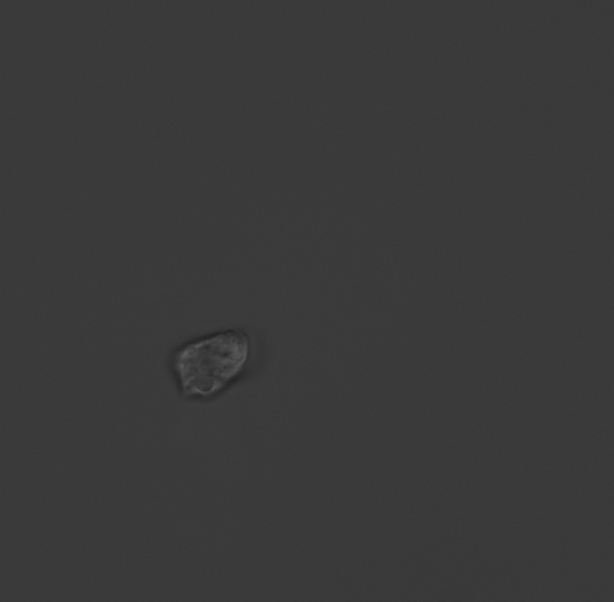

Supplement: S2 File — All the raw imagery/data used in this manuscript. (ZIP) [file pone.0261763.s002.zip › Raw Data/Figure 3 Imagery/b-left/Fig3b-2_t01.jpg]

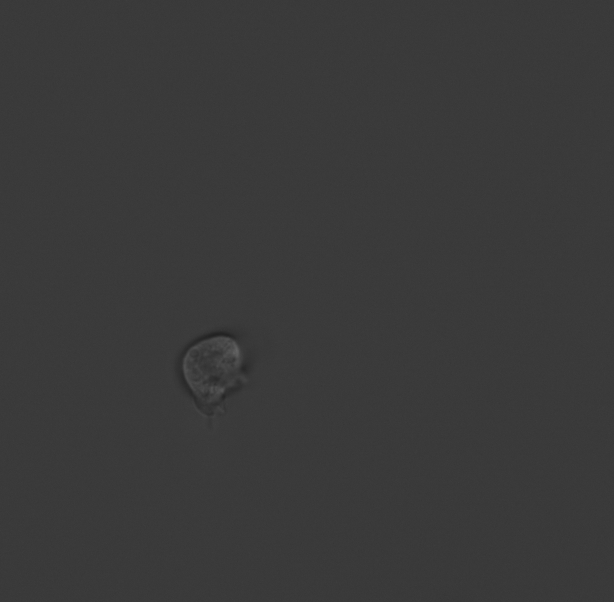

Supplement: S2 File — All the raw imagery/data used in this manuscript. (ZIP) [file pone.0261763.s002.zip › Raw Data/Figure 3 Imagery/b-left/Fig3b-2_t02.jpg]

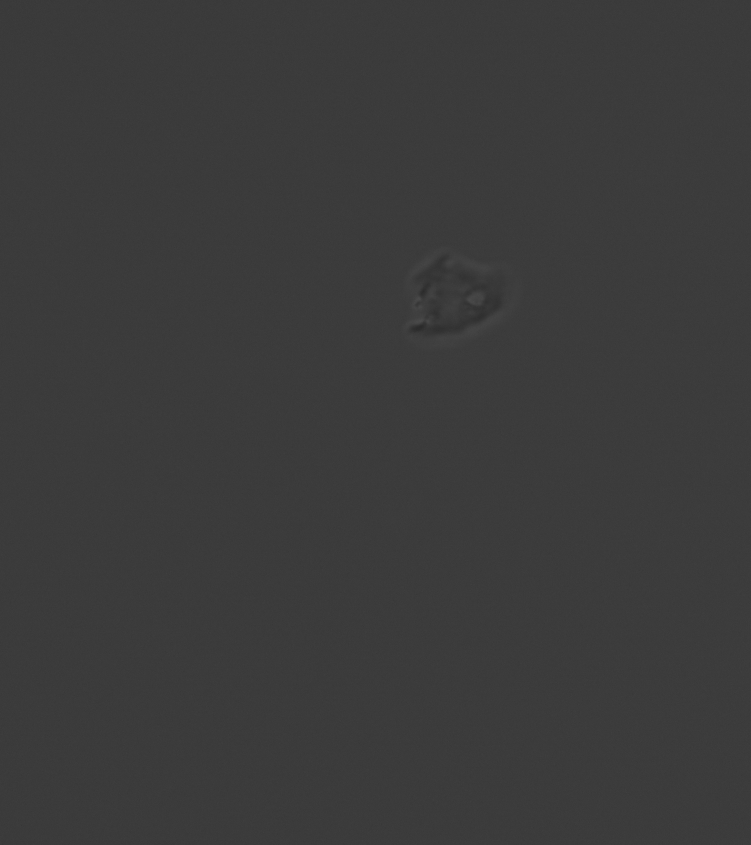

Supplement: S2 File — All the raw imagery/data used in this manuscript. (ZIP) [file pone.0261763.s002.zip › Raw Data/Figure 3 Imagery/b-right/Fig3b-3_t01.jpg]

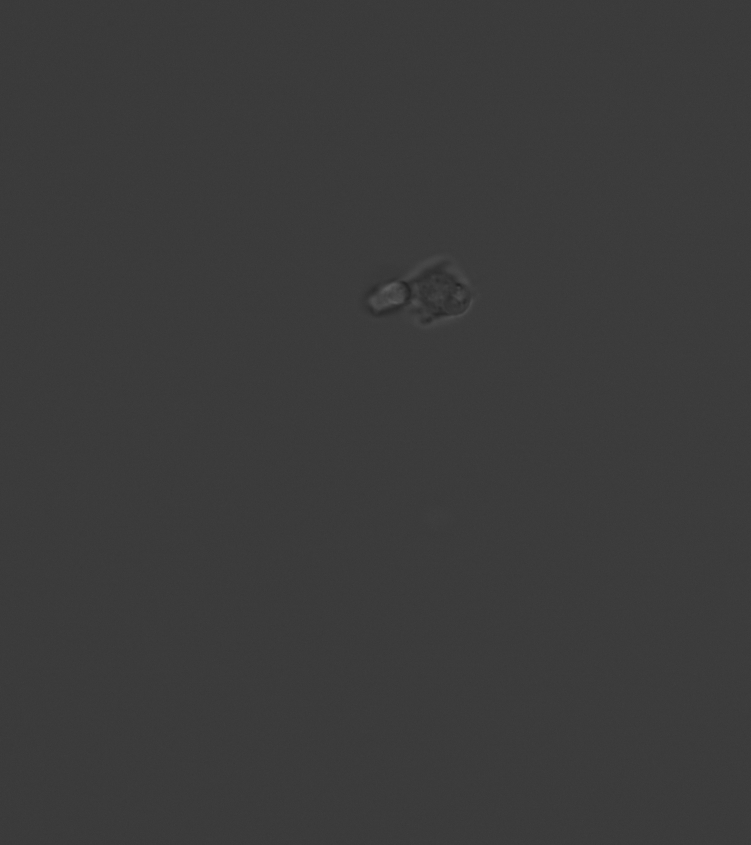

Supplement: S2 File — All the raw imagery/data used in this manuscript. (ZIP) [file pone.0261763.s002.zip › Raw Data/Figure 3 Imagery/b-right/Fig3b-3_t02.jpg]

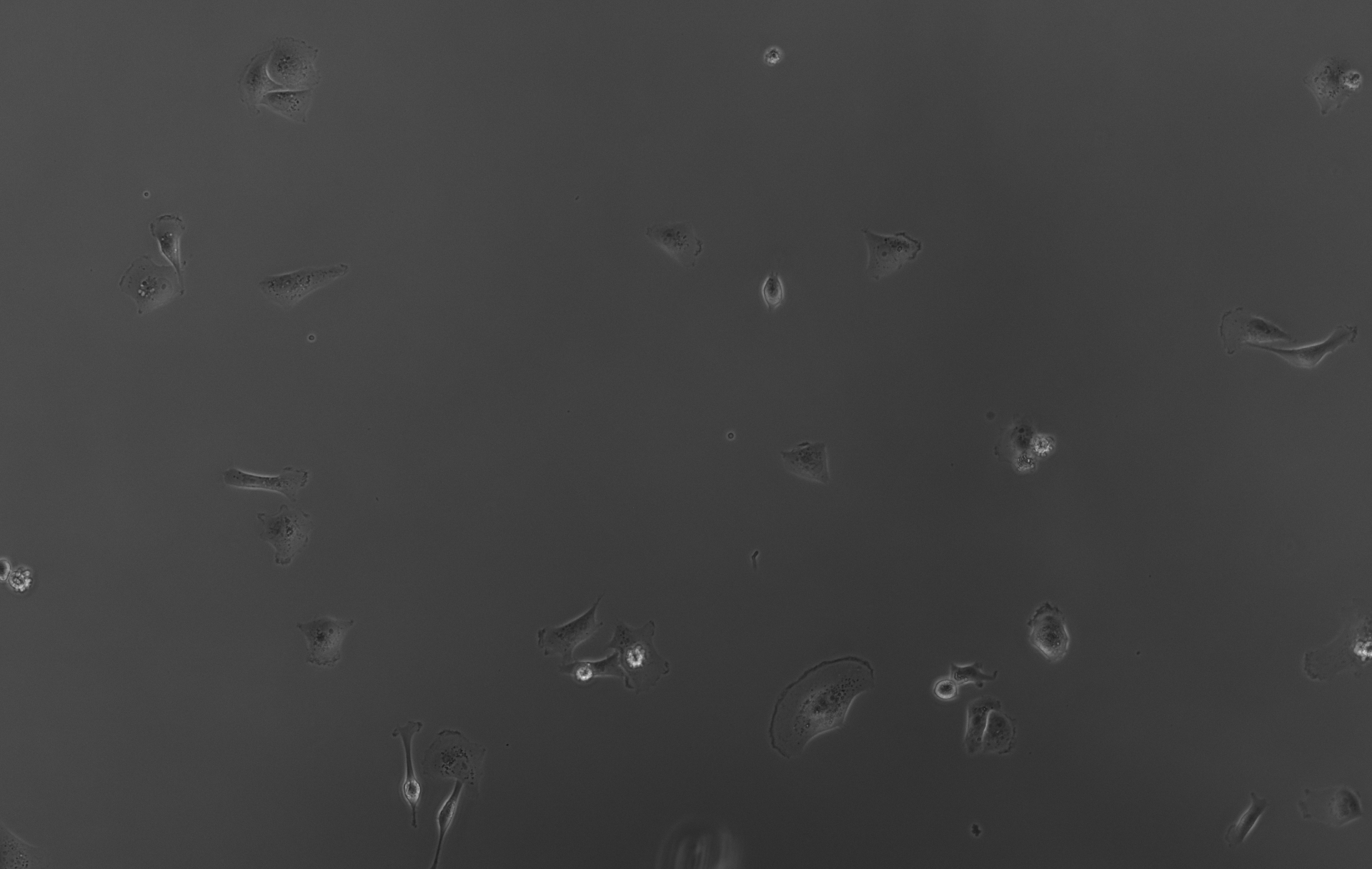

Supplement: S2 File — All the raw imagery/data used in this manuscript. (ZIP) [file pone.0261763.s002.zip › Raw Data/Figure 3 Imagery/c-left/Fig3c-1_t01.tif]

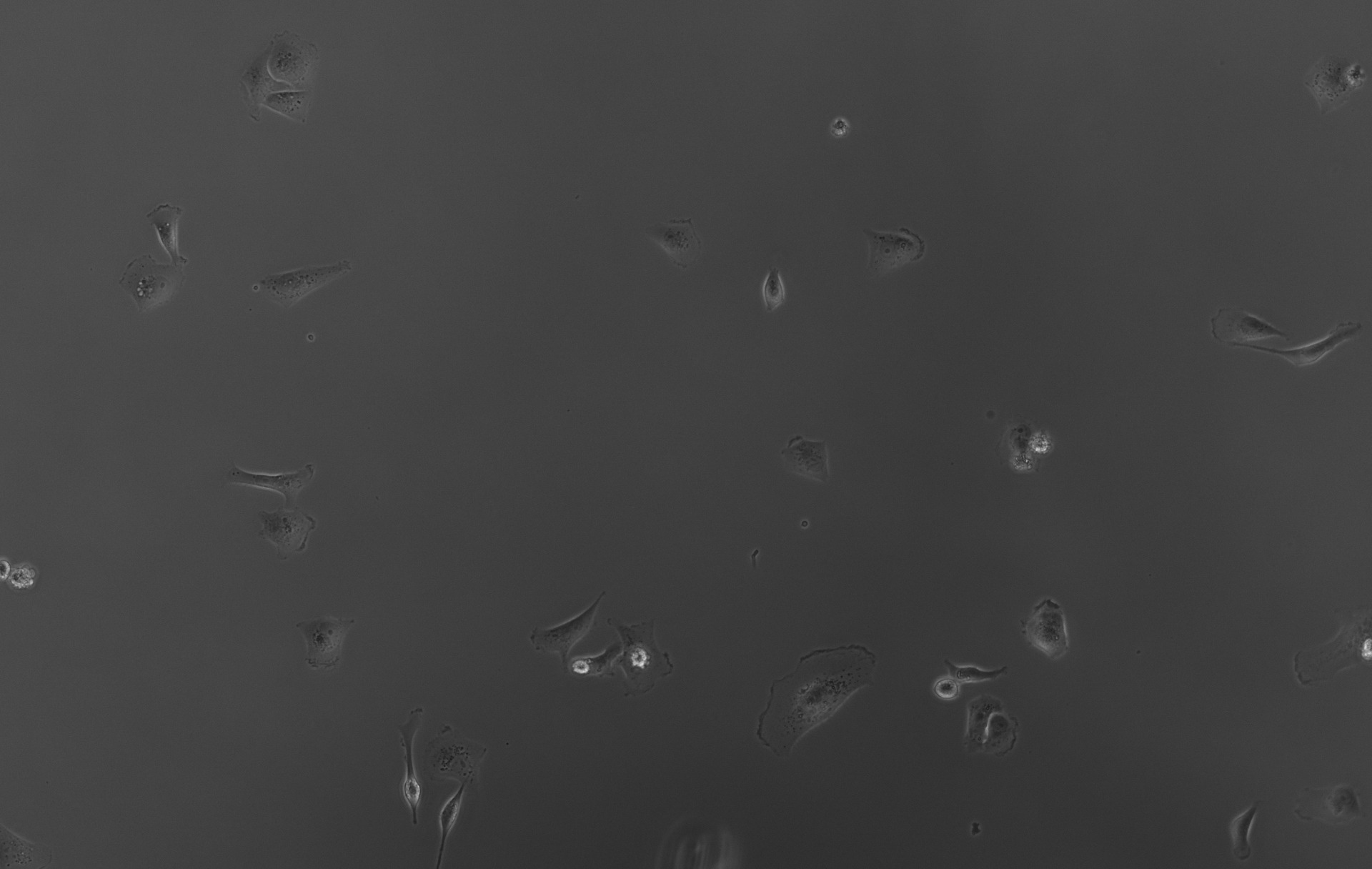

Supplement: S2 File — All the raw imagery/data used in this manuscript. (ZIP) [file pone.0261763.s002.zip › Raw Data/Figure 3 Imagery/c-left/Fig3c-1_t02.tif]

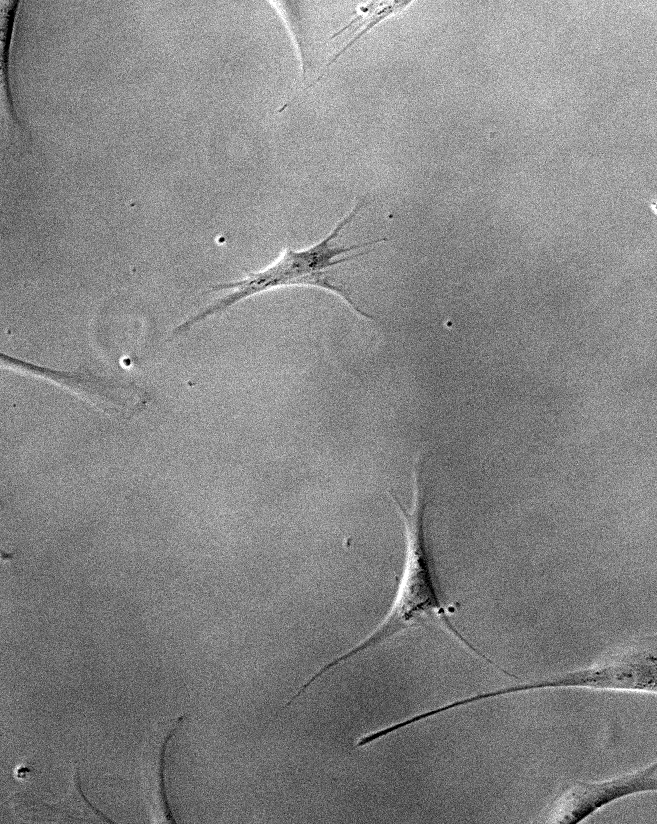

Supplement: S2 File — All the raw imagery/data used in this manuscript. (ZIP) [file pone.0261763.s002.zip › Raw Data/Figure 3 Imagery/d-left/Fig3d-1_t01.tif]

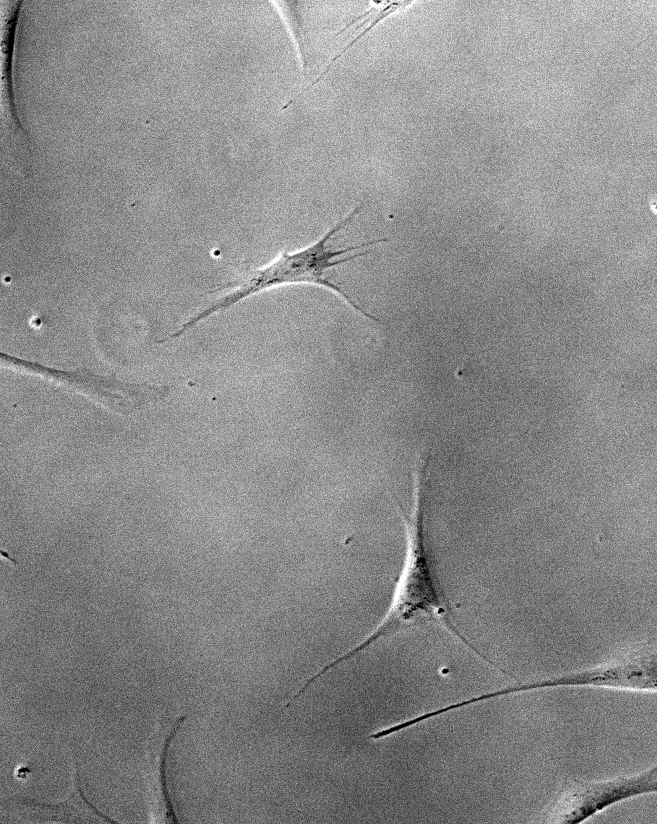

Supplement: S2 File — All the raw imagery/data used in this manuscript. (ZIP) [file pone.0261763.s002.zip › Raw Data/Figure 3 Imagery/d-left/Fig3d-1_t02.tif]

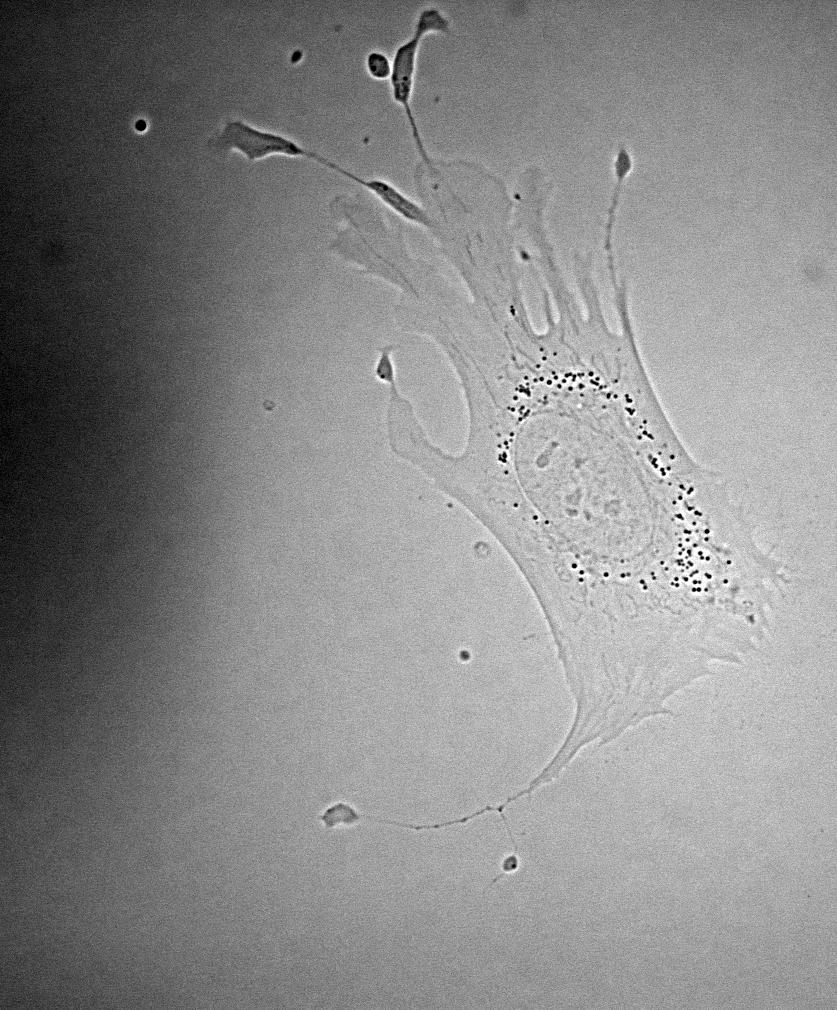

Supplement: S2 File — All the raw imagery/data used in this manuscript. (ZIP) [file pone.0261763.s002.zip › Raw Data/Figure 3 Imagery/d-right/Fig3d-2_t01.tif]

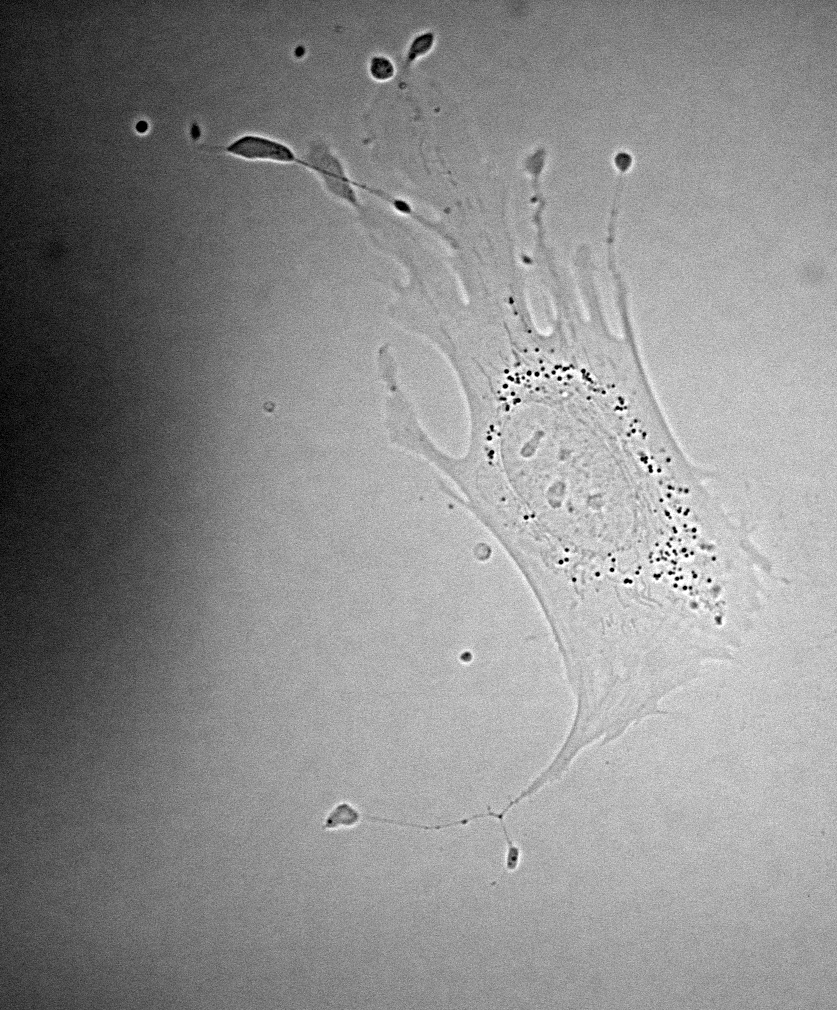

Supplement: S2 File — All the raw imagery/data used in this manuscript. (ZIP) [file pone.0261763.s002.zip › Raw Data/Figure 3 Imagery/d-right/Fig3d-2_t02.tif]

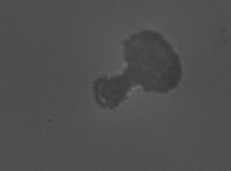

Supplement: S2 File — All the raw imagery/data used in this manuscript. (ZIP) [file pone.0261763.s002.zip › Raw Data/Figure 3 Imagery/e-left/Fig3e-1_t01.tif]

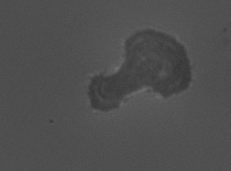

Supplement: S2 File — All the raw imagery/data used in this manuscript. (ZIP) [file pone.0261763.s002.zip › Raw Data/Figure 3 Imagery/e-left/Fig3e-1_t02.tif]

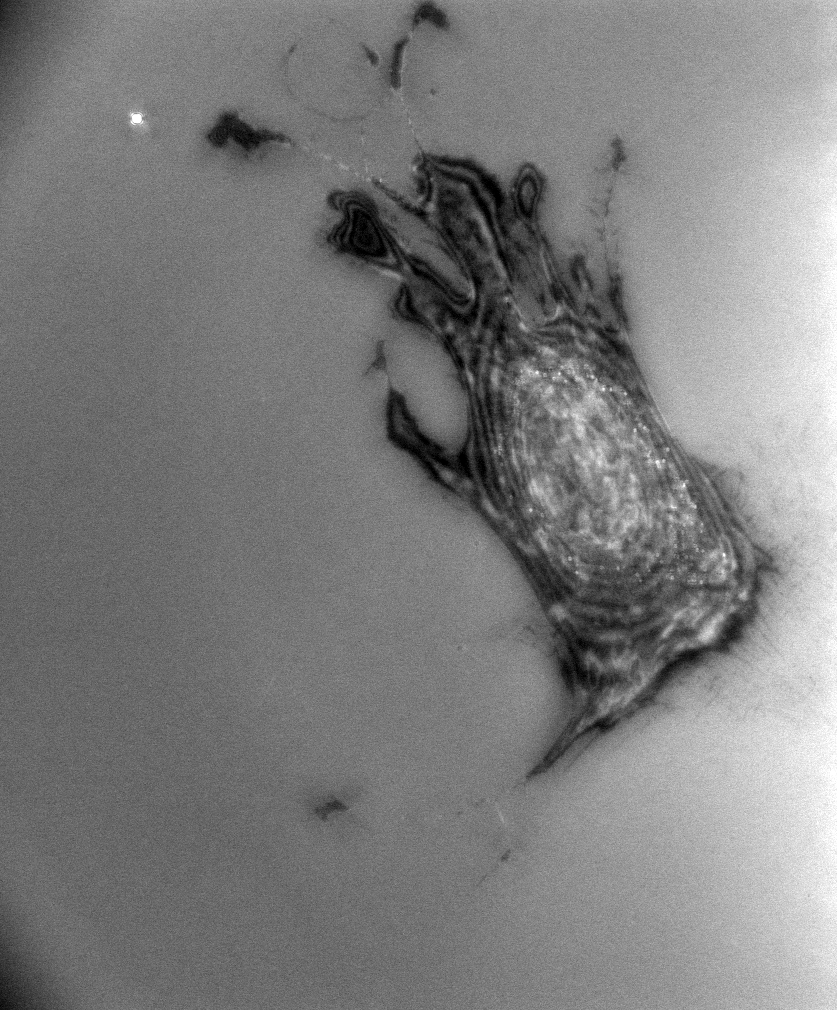

Supplement: S2 File — All the raw imagery/data used in this manuscript. (ZIP) [file pone.0261763.s002.zip › Raw Data/Figure 3 Imagery/e-right/Fig3e-2_t01.tif]

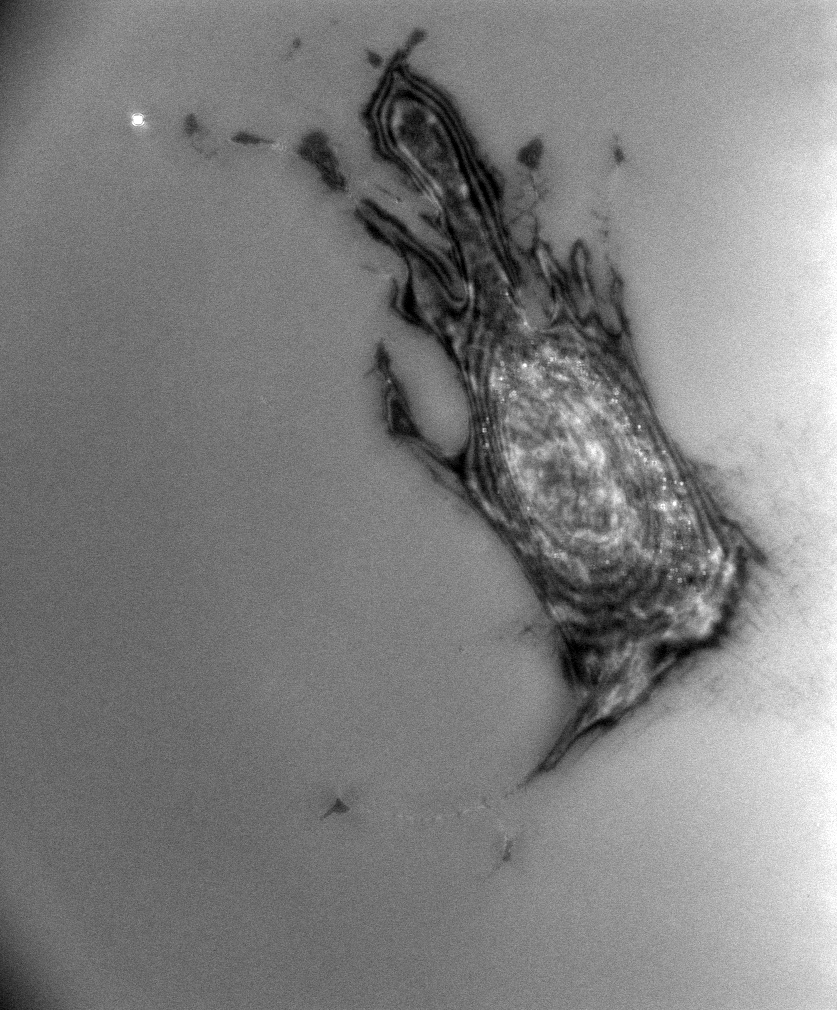

Supplement: S2 File — All the raw imagery/data used in this manuscript. (ZIP) [file pone.0261763.s002.zip › Raw Data/Figure 3 Imagery/e-right/Fig3e-2_t02.tif]

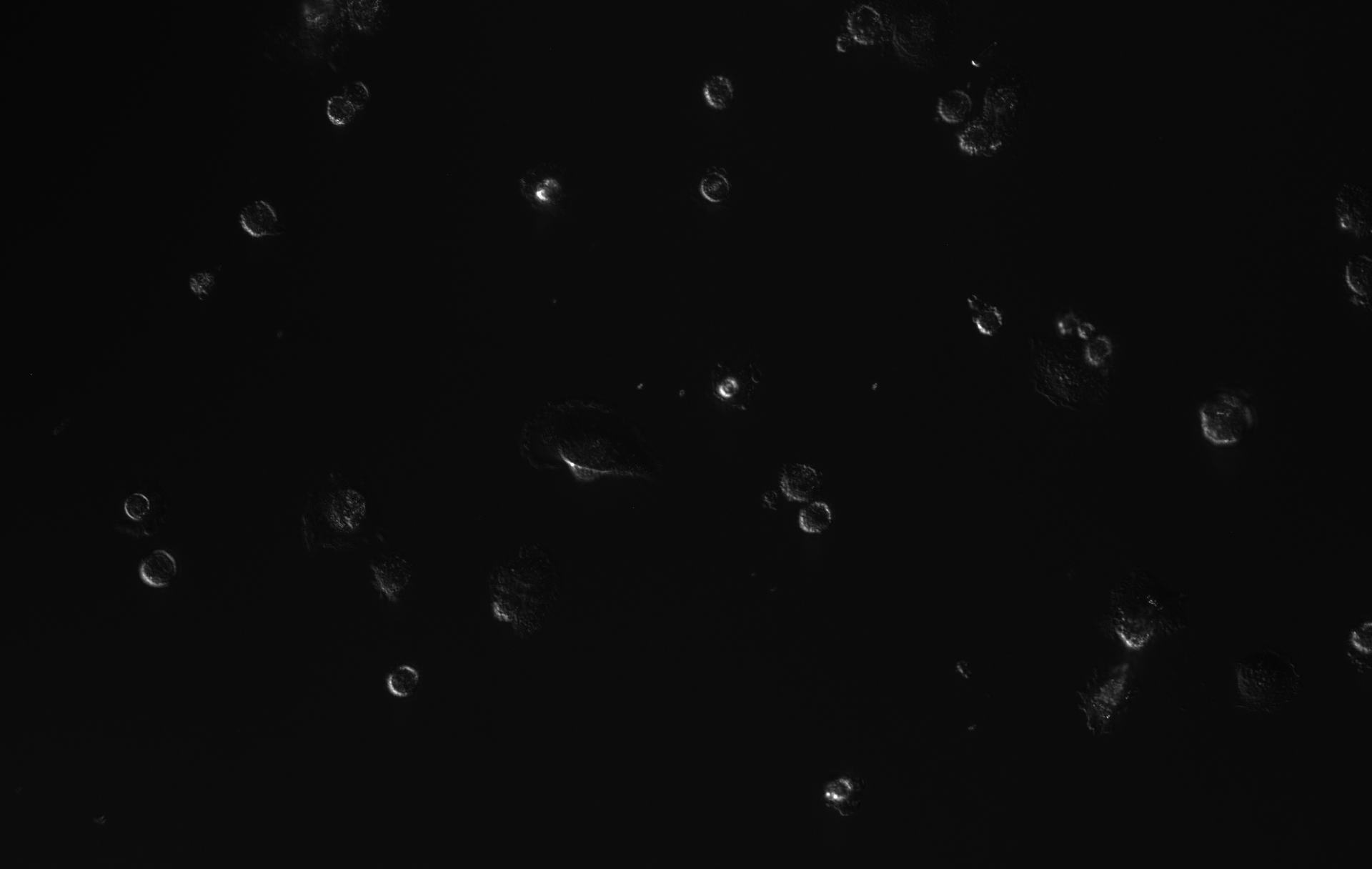

Supplement: S2 File — All the raw imagery/data used in this manuscript. (ZIP) [file pone.0261763.s002.zip › Raw Data/Figure 3 Imagery/f/Fig3f_t01.tif]

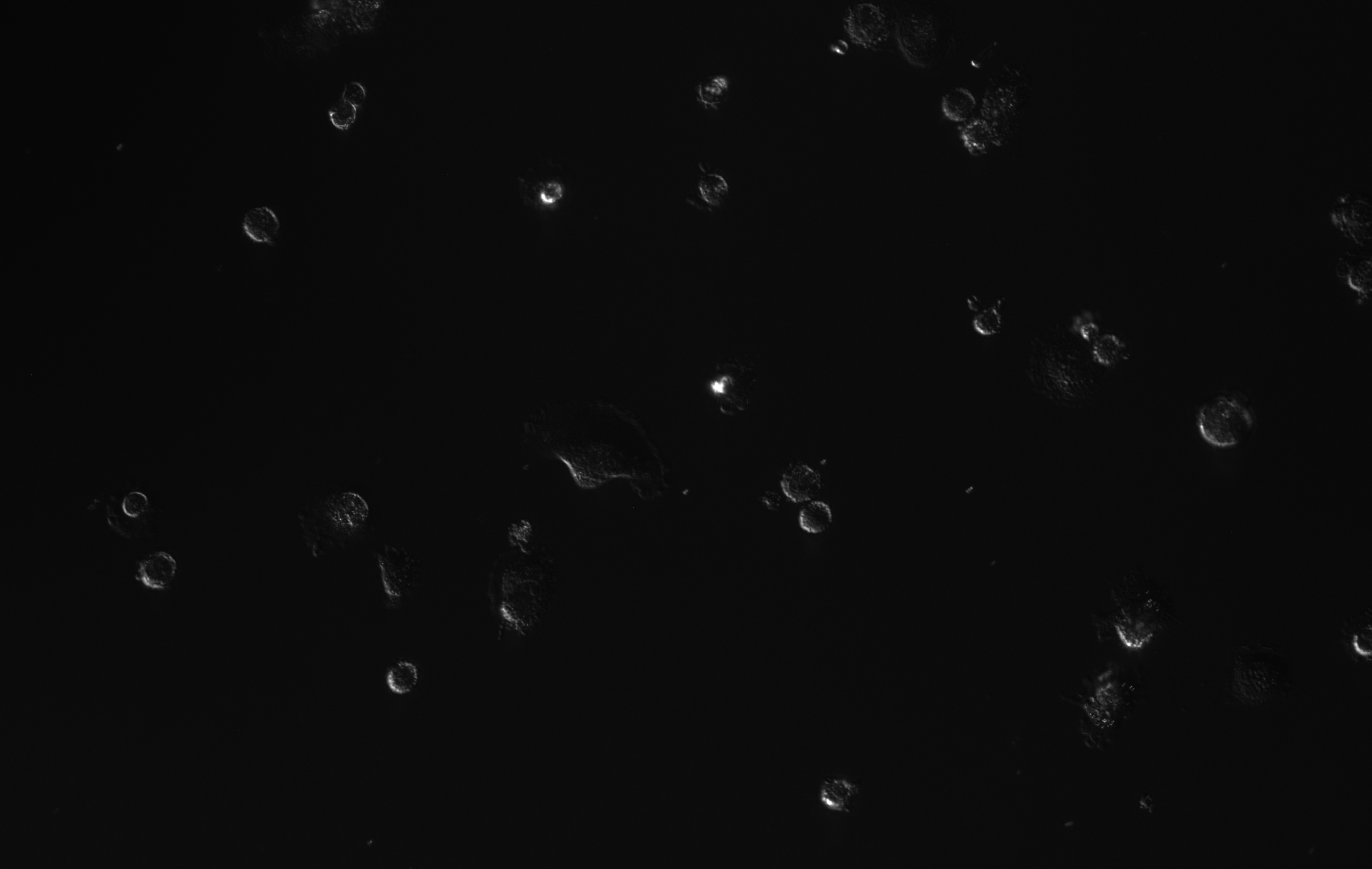

Supplement: S2 File — All the raw imagery/data used in this manuscript. (ZIP) [file pone.0261763.s002.zip › Raw Data/Figure 3 Imagery/f/Fig3f_t02.tif]

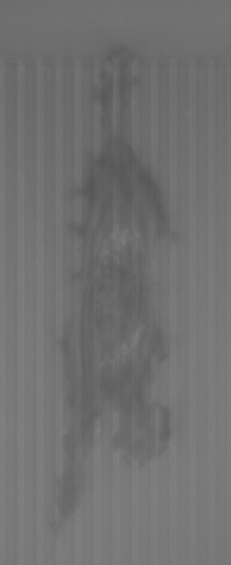

Supplement: S2 File — All the raw imagery/data used in this manuscript. (ZIP) [file pone.0261763.s002.zip › Raw Data/Figure 5 Imagery/a/Fig5a_t01.tif]

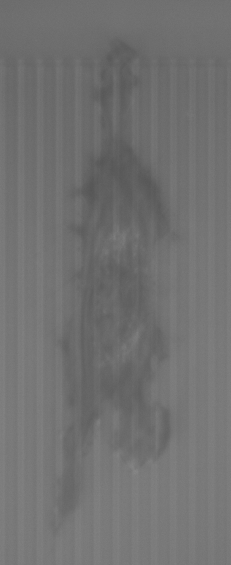

Supplement: S2 File — All the raw imagery/data used in this manuscript. (ZIP) [file pone.0261763.s002.zip › Raw Data/Figure 5 Imagery/a/Fig5a_t02.tif]

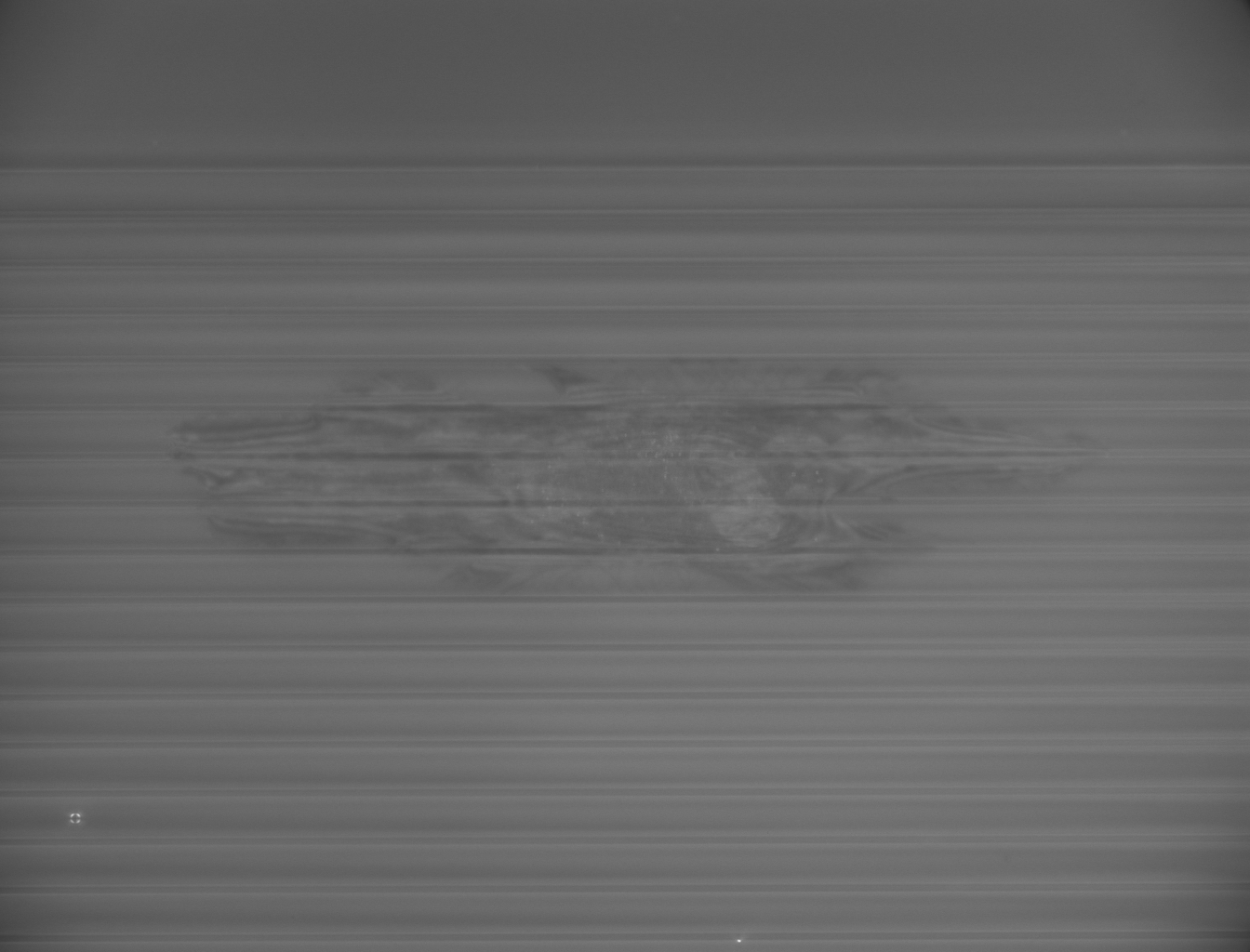

Supplement: S2 File — All the raw imagery/data used in this manuscript. (ZIP) [file pone.0261763.s002.zip › Raw Data/Figure 5 Imagery/b/Fig5b_t01.tif]

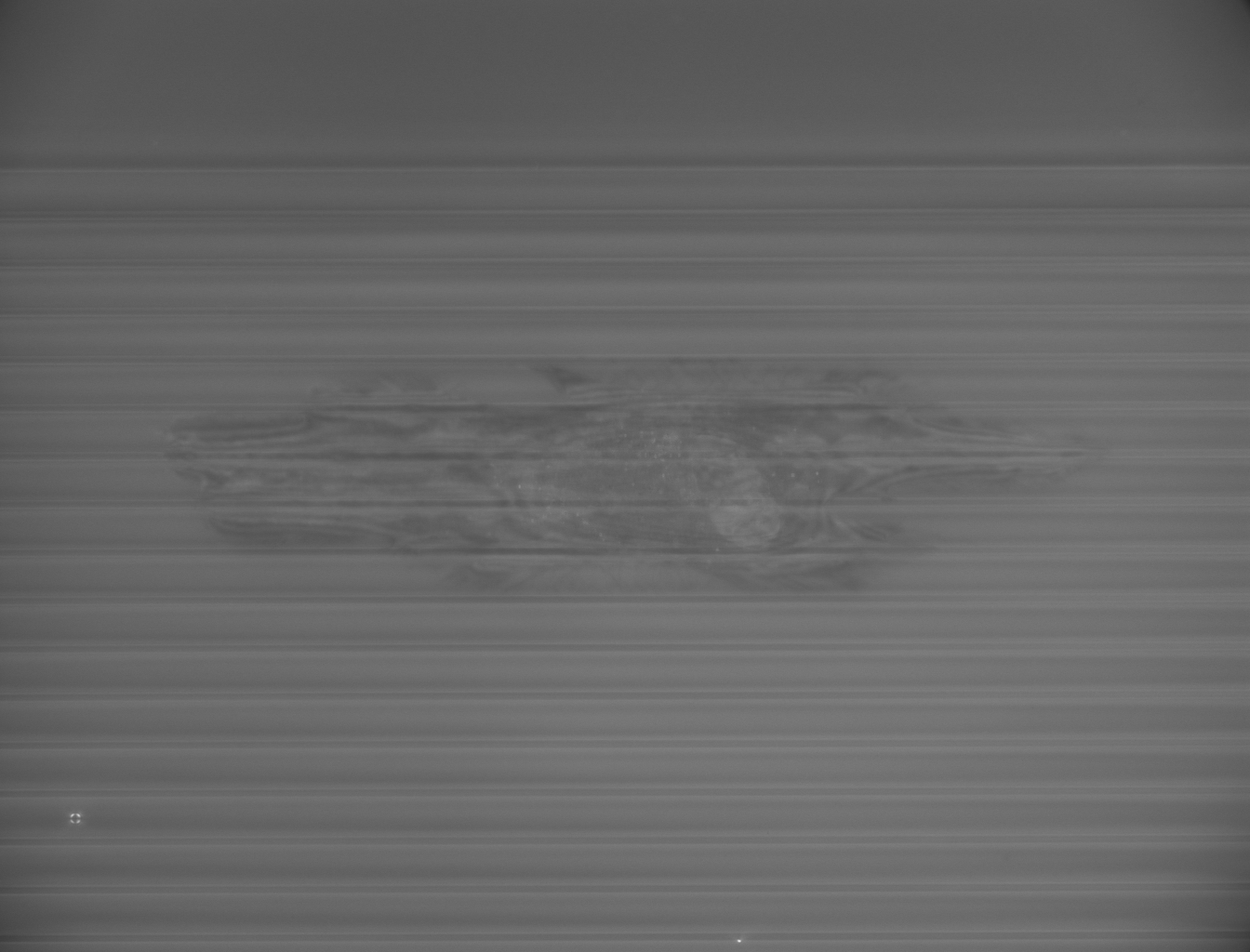

Supplement: S2 File — All the raw imagery/data used in this manuscript. (ZIP) [file pone.0261763.s002.zip › Raw Data/Figure 5 Imagery/b/Fig5b_t02.tif]
